# Exploring the characteristics, methods and reporting of systematic reviews with meta-analyses of time-to-event outcomes: a meta-epidemiological study

**DOI:** 10.1186/s12874-024-02401-4

**Published:** 2024-11-25

**Authors:** Marius Goldkuhle, Caroline Hirsch, Claire Iannizzi, Ana-Mihaela Zorger, Ralf Bender, Elvira C. van Dalen, Lars G. Hemkens, Ina Monsef, Nina Kreuzberger, Nicole Skoetz

**Affiliations:** 1 Institute of Public Health, Faculty of Medicine and University Hospital Cologne, University of Cologne, Kerpener Str. 62, 50937 Cologne, Germany; 2 Department of Medical Biometry, Institute for Quality and Efficiency in Health Care, Im Mediapark 8, D-50670 Cologne, Germany; 3 Princess Máxima Center for Pediatric Oncology, Heidelberglaan 25, Utrecht, 3584CS The Netherlands; 4 Research Center for Clinical Neuroimmunology and Neuroscience Basel (RC2NB), University Hospital Basel and University of Basel, Basel, Switzerland; 5 Department of Clinical Research, University Hospital Basel, University of Basel, Basel, Switzerland; 6 Meta-Research Innovation Center at Stanford (METRICS), Stanford University, Stanford, CA USA; 7 Meta-Research Innovation Center Berlin (METRIC-B), Berlin Institute of Health, Berlin, Germany

**Keywords:** Systematic review, Meta-analysis, Time-to-event outcomes, Survival analysis, Reporting quality, Quantitative analysis

## Abstract

**Background:**

Time-to-event analysis is associated with methodological complexities. Previous research identified flaws in the reporting of time-to-event analyses in randomized trial publications. These hardships impose challenges for meta-analyses of time-to-event outcomes based on aggregate data. We examined the characteristics, reporting and methods of systematic reviews including such analyses.

**Methods:**

Through a systematic search (02/2017-08/2020), we identified 50 Cochrane Reviews with ≥ 1 meta-analysis based on the hazard ratio (HR) and a corresponding random sample (*n* = 50) from core clinical journals (Medline; 08/02/2021). Data was extracted in duplicate and included outcome definitions, general and time-to-event specific methods and handling of time-to-event relevant trial characteristics.

**Results:**

The included reviews analyzed 217 time-to-event outcomes (Median: 2; IQR 1–2), most frequently overall survival (41%). Outcome definitions were provided for less than half of time-to-event outcomes (48%). Few reviews specified general methods, e.g., included analysis types (intention-to-treat, per protocol) (35%) and adjustment of effect estimates (12%). Sources that review authors used for retrieval of time-to-event summary data from publications varied substantially. Most frequently reported were direct inclusion of HRs (64%) and reference to established guidance without further specification (46%). Study characteristics important to time-to-event analysis, such as variable follow-up, informative censoring or proportional hazards, were rarely reported. If presented, complementary absolute effect estimates calculated based on the pooled HR were incorrectly calculated (14%) or correct but falsely labeled (11%) in several reviews.

**Conclusions:**

Our findings indicate that limitations in reporting of trial time-to-event analyses translate to the review level as well. Inconsistent reporting of meta-analyses of time-to-event outcomes necessitates additional reporting standards.

**Supplementary Information:**

The online version contains supplementary material available at 10.1186/s12874-024-02401-4.

## Background

Systematic reviews of time-to-event analyses, also referred to as survival analyses, provide fundamental evidence in many fields of research [1]. In randomized controlled trials (RCTs) and non-randomized studies alike, time-to-event outcomes combine the occurrence of events with information about how long they took to occur, considering the observation time of participants with and without an event (censored observations). Particularly relevant for medical research, e.g., in oncology or cardiology, this allows analyzing longer-term outcomes or outcomes which might occur in all participants at some point, such as death [2, 3]. Prominent measures of time-to-event data include Kaplan and Meier survival plots and probabilities, and the hazard ratio (HR) for between-group comparisons [1, 4]. For trial-level data, HRs are commonly calculated using the Cox proportional hazards regression model, which allows the consideration of relevant covariates [4]. Aggregate data meta-analyses pool HRs from individual RCTs.

Common methods of time-to-event analysis assume non-informative censoring, requiring that the distributions of event times and censoring times do not provide any information about each other [4]. In many circumstances, this is most vividly explained by the less restrictive assumption of random censoring: censoring should occur as if those censored were randomly drawn, which is questionable, for instance, when censoring occurs for adverse events or other reasons related to the intervention [2, 4, 5]. The Cox model assumes at least approximate proportionality of the hazards of the compared groups over time [4, 6, 7]. If violated, the HR is best interpreted as an average over the observed period, which might affect the between-study heterogeneity in meta-analyses of trials with variable follow-up durations [8]. Other challenges include competing events (like death) that may preclude the event of interest [9, 10], treatment switching [11, 12] and the poor reporting in trial publications [13–18]. Furthermore, the HR as relative effect measure is not always straightforward to interpret and might endanger exaggerated interpretation of treatment effects [19, 20]. This is why leading organizations in evidence synthesis recommend complementary absolute effect estimates, e.g., natural frequencies, risk differences or the number-needed-to-treat, calculated based on the pooled HR, as additional presentation of results [21–24].

To foster prioritization of remedial actions and further research, this meta-epidemiological analysis explored characteristics, reporting and methods of systematic reviews including time-to-event meta-analyses per review and per analyzed time-to-event outcome.

## Methods

We followed the Preferred Reporting Items for Systematic Reviews and Meta-Analyses (PRISMA) checklist´s adaption to meta-epidemiological research [25]. The project is registered under: osf.io/5qxbd.

### Eligibility criteria

We included systematic reviews of interventions with at least one pairwise meta-analysis based on aggregate data from RCTs. Reviews must have meta-analyzed a minimum of two trials evaluating a health-related time-to-event outcome using the HR. We considered any peer-reviewed full-text review published in English on any intervention type, medical field or setting. We excluded network meta-analyses, previous versions of updated reviews and co-publications of Cochrane reviews (CR).

### Identification and selection of reviews and studies

We chose an overall sample size of 100 reviews, 50 CR and 50 Non-Cochrane reviews (nCR), to represent a diverse and methodologically up-to-date landscape of systematic reviews. We systematically selected CR according to our inclusion criteria in a backwards fashion starting with publications in August 2020 from the Cochrane Database of Systematic Reviews. To identify a representative sample of nCR published during the publication timeframe corresponding to all eligible CR (28/02/2017 to 18/08/2020), an experienced information specialist (IM) developed a search strategy for Medline/Ovid (Table A1, Additional File 1; search date: 08/02/2021). To ensure the relevance of the included nCR, we limited our search to systematic reviews published in Core Clinical Journals, as defined by the U.S National Library of Medicine [26].

Titles and abstracts were screened and potentially eligible reviews selected based on their full text publications by two authors (MG, NK) independently. Discrepancies were resolved by discussion and, if necessary, by involving a third author (NS). We identified more potentially eligible nCR and selected, stratified by publication year, a random sample of 50 nCR published within the publication time of the CR. The random selection was performed by a third author not involved in the selection of reviews and extraction of data by using a random numbers generator.

Our assessment was limited to meta-analyses of outcomes under the main review comparison, which was in case of multiple comparisons the first comparison reported in the abstract. We did not assess any subgroup or sensitivity analyses. When the total and subtotal results for an individual eligible meta-analysis were reported, we extracted data on the total summary measure only. For feasibility, we excluded comparisons with one or more time-to-event meta-analyses that included more than 20 trials.

### Data extraction

All extractions were performed in duplicate by two authors (NK, CI, CH, AB, MG) (25 reviews extracted independently, 75 reviews double-checked after sufficient agreement). The data extraction sheet was developed a priori, piloted, and implemented on the data management platform Ninox (ninox.com). It was developed based on experience from previous methodological surveys of systematic reviews and based on published proposals for the reporting of time-to-event analyses in study publications [14, 15, 20, 27]. Any potential discrepancies were resolved by discussion and, where necessary, by involving a third author (NS). We provide our complete extraction sheets, including all items, their descriptions and response options in the appendix (Table A2, Additional File 1).

### Statistical analysis

Data are analyzed descriptively by means of absolute and relative frequencies for categorical data and medians, means and variability measures for continuous data.

We anticipated considerable differences in the handling of time-to-event specific trial characteristics between systematic review types (CR vs. nCR) and between different outcomes. Cochrane reviews are published under strict accordance with the Cochrane Handbook and without wording restrictions [22]. Because of these distinctions, we report our findings stratified by CR and nCR, even though our primary intent is not to compare review types. In addition, we present our findings stratified into three outcome categories in appendix tables that extend the data included in the text: Overall survival/all-cause mortality, composite outcomes including all-cause mortality and outcomes not including all-cause mortality, because of their different susceptibility to competing events, measurement robustness and relative importance.

## Results

We provide a flow diagram (A3), a description of our search results (A4) and a list of all included reviews (A5) in the appendix (Additional File 1).

### Characteristics of included reviews

The majority of reviews were published in 2017 and most frequently addressed oncological research questions in adults at advanced clinical stages and compared biologics/drugs to biologics/drugs (Table 1; extended data in appendix-table A6, Additional File 1).

**Table 1:** Summary of review characteristics

Reviews		Overall (*N* = 100)	Cochrane (*n* = 50)	Non-Cochrane (*n* = 50)
Publication				
*Publication year*	2017	36% (36)	36% (18)	36% (18)
	2018	28% (28)	28% (14)	28% (14)
	2019	18% (18)	18% (9)	18% (9)
	2020	18% (18)	18% (9)	18% (9)
*Journal Impact Factor 2021*	Median (IQR)		11.87*	4.41 (3.33–6.18)
	Mean (Range)		11.87*	6.37 (1.817–35.86)
*Update of previous review*		27% (27)	50% (25)	4% (2)
*Multiple comparisons*		28% (28)	44% (22)	12% (6)
Population				
*Medical field*	Neoplasms	82% (82)	86% (43)	78% (39)
	Circulatory system	11% (11)	4% (2)	18% (9)
	Other	7% (7)	10% (5)	4% (2)
*Medical condition*	Breast cancer	13% (13)	10% (5)	16% (8)
	Colorectal cancer	9% (9)	8% (4)	10% (5)
	Non-small cell lung cancer	8% (8)	6% (3)	10% (5)
	Prostate cancer	6% (6)	12% (6)	0% (0)
	Other	7% (7)	62% (31)	64% (32)
*Age group* ^*#*^	Adults	96% (96)	98% (49)	94% (47)
Comparisons				
	Biologics/ drug vs. Biologics/ drug	37% (37)	24% (12)	50% (25)
	Surgical procedure vs. Surgical procedure	7% (7)	8% (4)	6% (3)
	Biologics/ drug vs. Supportive/ Optimal care	4% (4)	4% (2)	4% (2)
	Biologics/ drug vs. Observation	4% (4)	0% (0)	8% (4)
	Other	48% (48)	64% (32)	32% (16)
Outcomes				
*Planned outcomes*	Median (IQR)	5 (4–8)	7 (5–8)	4 (3–5)
	Mean (Range)	5.79 (1–15)	6.82 (3–12)	4.67 (1–15)
*TTE outcomes in methods*	Median (IQR)	2 (2–2)	2 (2–3)	2 (2–2)
	Mean (Range)	2.39 (1–12)	2.17 (1–4)	2.62 (1–12)
	OS, ACM or death from any cause	89% (89)	88% (44)	90% (45)
	Progression-free survival	44% (44)	36% (18)	52% (26)
	Disease-free survival	13% (13)	16% (8)	10% (5)
	Myocardial infarction	5% (5)	0% (0)	10% (5)
	Stroke	5% (5)	0% (0)	10% (5)
	Other^§^	72% (72)	74% (37)	70% (35)
	Unclear/ Not reported	5% (5)	2% (2)	3% (3)
*Outcomes analyzed*	Median (IQR)	5 (3–6)	5 (4–6,75)	4 (2,25–5)
	Mean (Range)	4.83 (1–12)	5.34 (1–12)	4.32 (1–12)
*TTE outcomes analyzed*	Median (IQR)	2 (1–2)	2 (1–2)	2 (2–2)
	Mean (Range)	2.23 (1–12)	1.92 (1–4)	2.54 (1–12)
	OS, ACM or death from any cause	89% (89)	84% (42)	94% (47)
	Progression-free survival	39% (39)	26% (13)	52% (26)
	Disease-free survival	10% (10)	10% (5)	10% (5)
	Myocardial infarction	6% (6)	0% (0)	12% (6)
	Stroke	5% (5)	0% (0)	10% (5)
	Other	78% (78)	70% (35)	86% (43)
*TTE outcome(s) in methods not among analyzed*		11% (11)	20% (10)	2% (1)
*Reasons for difference*	Outcome not in trial(s)	7% (7)	14% (7)	0% (0)
	No TTE data in trial(s)	2% (2)	2% (1)	0% (0)
	Not pooled due to heterogeneity	1% (1)	2% (1)	0% (0)
	Not reported	1% (1)	0% (0)	2% (1)
	Not applicable	89% (89)	80% (40)	98% (49)
Sample size				
*Included studies*	Median (IQR)	5 (4–8)	5 (3–10)	6 (4–8)
	Mean (Range)	6.69 (2–24)	7.12 (2–24)	6.26 (2–19)
*Total population*	Median (IQR)	1722 (97–4390)	1415 (572–4022)	1866 (1395–4526)
	Mean (Range)	3877 (170–56004)	2795 (170–13216)	4911 (343–56004)
	Not reported	12% (12)	14% (7)	10% (5)
*Studies in TTE-MA*	Median (IQR)	4 (3–7)	4 (2–6)	5 (4–7)
	Mean (Range)	5.25 (2–19)	5.05 (2–19)	5.40 (2–19)
*Population in TTE-MA*	Median (IQR)	811 (308–2876)	711 (177–2327)	1042 (698–3173)
	Mean (Range)	5745 (181–38723)	2656 (181–13949)^§^	8985 (482–38723)
	Not reported	23% (49)	8% (7)	34% (42)

Abbreviations IQR = interquartile range, MA = meta-analysis, TTE = time-to-event

* All CR share the impact factor of the Cochrane Database of Systematic Reviews; ^#^ One CR included children and adults, for three nCR no information was provided. ^§^ None of the analyzed outcomes under “Other” were used in more than four reviews. ^§^ This number exceeds the upper range of the total review population, since the review including the respective analysis did not report a total review population)

They included a median of five studies (interquartile range (IQR) 4–8) with a median of four studies (IQR 3–7) in time-to-event outcome analyses. The median overall population was 1722 participants (IQR 978–4390) with 811 participants (IQR 308–2876) in time-to-event meta-analyses.

### Analyzed time-to-event outcomes

Reviews reported overall a median of five (IQR 4–8) outcomes, containing a median of two (IQR 2–2) time-to-event outcomes in their methods sections. Median five (IQR 3–6) outcomes and a median (IQR 1–2) of two time-to-event outcomes were analyzed quantitatively in meta-analyses. In several reviews, not all time-to-event outcomes mentioned in the methods were analyzed, primarily because the included trials did not assess the respective outcome. The most frequently planned time-to-event outcomes were overall survival, all-cause death or death from any cause in 89% and progression-free survival in 39% of reviews. They were also the most commonly analyzed outcomes. The majority of reviews (69% (69/100 reviews); CR: 92% (46/50 reviews), nCR: 46% (23/50 reviews)) selected a time-to-event outcome as primary outcome, which commonly was overall survival, all-cause mortality or death from any cause (appendix-table A7, Additional File 1).

**Table 2:** Characteristics of time-to-event outcomes in reviews

			Reviews			TTE outcomes	
		Overall (*N* = 100)	Cochrane (*n* = 50)	Non-Cochrane (*n* = 50)	Overall (*N* = 217)	Cochrane (*n* = 93)	Non-Cochrane (*n* = 124)
*TTE outcome definitions provided*		55% (55)	80% (40)	30% (15)	48% (104)	83% (77)	22% (27)
*Composite TTE outcomes*	Yes	39% (39)	42% (21)	36% (18)	22% (47)	24% (22)	20% (25)
	Unclear/ Not reported	28% (28)	12% (6)	44% (22)	14% (30)	8% (7)	19% (23)
*Composite events described for composite outcome*		87% (34)	90% (19)	83% (15)	87% (41)	91% (20)	84% (21)
*All-cause death part of outcome*	Yes	91% (91)	86% (43)	96% (48)	57% (124)	65% (60)	52% (64)
	Unclear	31% (31)	14% (7)	48% (24)	14% (31)	8% (7)	19% (24)
*Death as competing event possible*	Yes	29% (29)	40% (20)	18% (9)	29% (64)	28% (26)	31% (38)
	Unclear	31% (31)	16% (8)	46% (23)	15% (32)	10% (9)	19% (23)
*Outcomes reporting as events or absence*	Absence of event only	61% (61)	48% (24)	74% (37)	54% (118)	53% (49)	56% (69)
	Event only	24% (24)	26% (13)	22% (11)	37% (81)	30% (28)	43% (53)
	Both (with reasoning)	5% (5)	10% (5)	0% (0)	5% (10)	11% (10)	0% (0)
	Both (without reasoning)	8% (8)	12% (6)	4% (2)	4% (8)	6% (6)	2% (2)
*Follow-up start in outcome definitions*	Randomization	32% (32)	48% (24)	16% (8)	32% (70)	57% (53)	14% (17)
	Allocated treatment	2% (2)	2% (1)	2% (1)	2% (4)	2% (2)	2% (2)
	Enrollment	2% (2)	2% (1)	0% (0)	3% (6)	6% (6)	0% (0)
	Not applicable (e.g., start of follow-up not reported)	62% (62)	46% (23)	82% (41)	62% (135)	32% (30)	85% (105)

Abbreviations ACM = all-cause mortality, HR = hazard ratio, IPD = individual participant data, OS = overall survival, TTE = time to event

Overall, the reviews analyzed 217 individual time-to-event outcomes (Table 2; extended data in appendix-table A7, Additional File 1). If grouped by outcome type, 41% (89/217 outcomes) of analyzed outcomes solely addressed the outcome event all-cause death, i.e., overall survival or all-cause mortality, 29% (63/217 outcomes) were composite outcomes that included all-cause death as outcome event, e.g., progression-free survival, and 30% (65/217 outcomes) were outcomes that did not include all-cause death. Outcome definitions were provided for less than half (48% (104/217 outcomes); CR: 83% (77/93 outcomes), nCR: 22% (27/124 outcomes)) of all review time-to-event outcomes. 22% (47/217 outcomes) of outcomes were composite outcomes for which composing events were commonly reported. Starting points of follow-up for individual outcomes were not regularly defined, but if they were, it most often was randomization.

Results of time-to-event meta-analyses were typically (54% (118/217 outcomes)) reported as absence of event only, e.g., survival, as opposed to reporting of results as events, e.g., death. Some review authors reported the same outcome inconsistently as event and absence thereof, few explained their decision. Outcomes not including all-cause death were most frequently reported as events (appendix-table A7, Additional File 1).

### General methodological characteristics of reviews and their time-to-event outcome analyses

Less than half (43% (43/100 reviews); CR: 72% (36/50 reviews), nCR: 14% (7/50 reviews)) of reviews reported the primary trial analysis (e.g., intention-to-treat, per protocol, etc.) that was preferred for meta-analysis (appendix-table A8, Additional File 1). Review authors preferred analyses according to the intention-to-treat approach in all cases. The actually included analysis principles, also commonly per intention-to-treat, were reported in 38% (38/100 reviews; CR: 54% (27/50 reviews), nCR: 22% (11/50 reviews)) of reviews. Few reviews (15% (15/100 reviews); CR: 26% (13/50 reviews), nCR: 4% (2/100 reviews)) reported if they used adjusted or unadjusted effect estimates from trials for meta-analyses. The most frequent options were eligibility of both, analyses adjusted for covariates or unadjusted analyses only. Handling divergently adjusted effects was sporadically reported: e.g., as potential source of heterogeneity between trials or as combined in the same analysis, and discrepancies in adjustment of effects were seldomly mentioned. None of the reviews compared covariate adjusted and unadjusted trial estimates.

To address statistical heterogeneity between trials, review authors commonly reported subgroup analyses or a random-effects meta-analysis (Table 3). Time-to-event data was most frequently pooled with random-effects models and the inverse variance method.

**Table 3:** General methodological characteristics of time-to-event outcome meta-analyses in reviews

Reviews		Overall (*N* = 100)	Cochrane (*n* = 50)	Non-Cochrane (*n* = 50)
Methods to pool time-to-event data specified in review methods				
*Random-effects model*	Inverse variance	38% (38)	48% (24)	28% (14)
	Hartung-Knapp-Sidik-Jonkman	2% (2)	0 (0%)	2% (2)
	Not reported	6% (6)	4% (2)	8% (4)
*Fixed-effect model*	Inverse variance	7% (7)	12% (6)	2% (1)
	Other (Peto or Mantel-Haenszel)	3% (3)	4% (2)	2% (1)
	Not reported	1% (1)	0% (0)	2% (1)
*Either*,* depending on heterogenity*	Inverse variance	15% (15)	18% (9)	12% (6)
	Mantel-Haenszel or inverse variance	3% (3)	0% (0)	6% (3)
	Not reported	22% (22)	22% (11)	22% (11)
*Both (e.g.*,* one as sensitivity analysis)*	Inverse variance	5% (5)	4% (2)	6% (3)
	Other*	2% (2)	0% (0)	4% (2)
	Not reported	5% (5)	6% (3)	4% (2)
*Model and method not reported*		2% (2)	2% (1)	2% (1)
*Heterogeneity parameter specification* ^*#*^	DerSimonian-Laird	31% (27)	22% (9)	39% (18)
	Paule-Mandel	2% (2)	0%	4% (2)
	Not reported	66% (58)	78% (32)	57% (26)
Methods to pool time-to-event data used in results (e.g., indicated in forest plot)				
*Random effects*	Inverse variance	57% (57)	72% (36)	42% (21)
	Not reported	11% (11)	0% (0)	22% (11)
*Fixed-effect*	Inverse variance	24% (24)	24% (12)	24% (12)
	Peto	2% (2)	2% (1)	2% (1)
	Not reported	4% (4)	0% (0)	8% (4)
*Both (e.g.*,* one as sensitivity analysis)*	Inverse variance	4% (4)	2% (1)	6% (3)
	Not reported	2% (2)	0% (0)	4% (2)
*Model and method not reported*		1% (1)	2% (1)	0% (0)
Handling of heterogeneity				
*Heterogeneity handling per review*	Subgroup analyses	59% (59)	80% (40)	38% (19)
	Random-effects meta-analysis performed	55% (55)	54% (27)	56% (28)
	No pooling if too heterogeneous	12% (12)	24% (12)	0% (0)
	Meta-regression	7% (7)	4% (2)	10% (5)
	Unclear/ Not reported	15% (15)	2% (2)	26% (13)
*Heterogeneity handling per outcome*	Subgroup analyses	9% (9)	16% (8)	2% (1)
	Subgroup analyses and meta-regression	2% (2)	4% (2)	0% (0)
	Meta-regression	1% (1)	0% (0)	2% (1)
	Shared frailty survival model (based on IPD)	1% (1)	0% (0)	2% (1)
	Not reported per outcome	87% (86)	80% (40)	94% (47)

Abbreviations HR = hazard ratio IPD = individual participant data, MA = meta-analysis

* Other includes one review that reported the use of the inverse variance method under a random effects model and, in case of available data (O-E and variance) in trial publications, the Peto’s method for a fixed-effects model. It includes a second review that reported the use of the inverse variance method for random-effects meta-analysis and the Mantel-Haenzel method for a separate fixed-effects meta-analysis; ^#^ Applies to 87 reviews (CR: 41; nCR: 46) which reported to perform a random-effects model in their methods, either alone, depending on the degree of heterogeneity or together with a fixed effects model

### Time-to-event specific methods used in the included systematic reviews

Methods to obtain time-to-event summary data from included trials varied substantially (Table 4; extended data in appendix-table A9, Additional File 1). In 36% (18/50) of nCR no approaches to retrieve time-to-event data were reported. The most frequently used methods were direct inclusion of available HRs in 64% (64/100 reviews), particular sets of methods, e.g., those collected by Tierney [1] or Parmar [28], in 46% (46/100 reviews), and available log(HR)s with a standard error in 16% (16/ 100 reviews) of reviews. Data sources, if reported for individual outcomes, were most frequently HRs and confidence intervals, P-values with other information and survival curves. In total, 63% (63/100) of reviews reported at least one approach to recalculate summary time-to-event data from trial publications, in contrast to 16% (16/100) reviews that reported only HRs and confidence intervals. For individual review outcomes, 18% (18/100) of reviews reported any approach to recalculate summary time-to-event data. Review authors who reported direct inclusion of a HR or log(HR), most often did not provide further specifications of the eligible HR. Sporadic specifications include HRs from Cox models, log-rank tests or survival curves or HRs recalculated from median survival times.

**Table 4:** Time-to-event specific methods applied in reviews

			Reviews			TTE outcomes	
		Overall (*N* = 100)	Cochrane (*n* = 50)	Non-Cochrane (*n* = 50)	Overall (*N* = 217)	Cochrane (*n* = 93)	Non-Cochrane (*n* = 124)
*HR type eligible in reviews*	HR/ log(HR) not further specified	91% (91)	94% (47)	88% (44)	NA	NA	NA
	HR/ log(H)R from Cox model	2% (2)	4% (2)	0% (0)	NA	NA	NA
	HR / log(HR) from Cox model, log-rank test and Kaplan Meier Curve	1% (1)	2% (1)	0% (0)	NA	NA	NA
	Not reported	6% (6)	0% (0)	12% (6)	NA	NA	NA
*HR types eligible per outcome*	HR/ log(HR) from Cox model	1% (1)	2% (1)	0% (0)	0% (1)	1% (1)	0% (0)
	HR / log(HR) from median survival times and confidence intervals	1% (1)	2% (1)	0% (0)	0% (1)	1% (1)	0% (0)
	Unclear/ Not reported	98% (98)	96% (48)	100% (50)	99% (215)	98% (91)	100% (124)
*Methods to obtain TTE data per review*	HR and confidence intervals	64% (64)	66% (33)	62% (31)	NA	NA	NA
	Specified set of methods (e.g., Tierney 2007, Cochrane Handbook, etc.)	46% (46)	76% (38)	16% (8)	NA	NA	NA
	log(HR) and standard error	16% (16)	26% (13)	6% (3)	NA	NA	NA
	Survival curves	13% (13)	14% (7)	12% (6)	NA	NA	NA
	HR with other information (e.g., events per arm, total events, etc.)	10% (10)	16% (8)	4% (2)	NA	NA	NA
	P-value with additional information (e.g., total events, etc.)	8% (8)	8% (4)	8% (4)	NA	NA	NA
	Other*	11% (11)	14% (7)	8% (4)	NA	NA	NA
	Unclear/ Not reported	22% (22)	8% (4)	36% (18)	NA	NA	NA
*Recalculation of TTE data reported for outcome*		18% (18)	34% (17)	2% (1)	NA	NA	NA
*Methods to obtain TTE data for outcome*	HR and confidence intervals	9% (9)	18% (9)	0% (0)	7% (16)	17% (16)	0% (0)
	P-value together with additional information (e.g., events, etc.)	4% (4)	8% (4)	0% (0)	5% (10)	11% (10)	0% (0)
	Survival curves	7% (7)	12% (6)	2% (1)	5% (10)	10% (9)	1% (1)
	Other^#^	6% (6)	12% (6)	0% (0)	5% (11)	12% (11)	0% (0)
	Unclear/ Not reported	90% (90)	80% (40)	100% (50)	89% (193)	75% (70)	99% (123)

Abbreviations ACM = all-cause mortality, HR = hazard ratio, IPD = individual participant data, OS = overall survival, TTE = time to event

* Other includes, for example, individual participant data (recalculated or from publication), median survival times and time-point specific survival times; ^#^ Other includes, for example, HR with other information (e.g., events per arm, total events, etc.), time-point specific survival times, IPD recalculated or from publication and median survival times

Risk of bias assessments were predominately performed with the Cochrane Risk of Bias 1 tool on study level. Time-to-event specific risk of bias criteria were applied in four CR, addressing informative censoring and competing events. Grading of Recommendations Assessment, Development and Evaluation (GRADE) certainty of the evidence ratings were included in all CR and 14% (7/50 reviews) of nCR, none of which described criteria one could consider time-to-event specific. 45% of all assessed time-to-event outcomes were included in GRADE Summary of Findings tables.

### Results of meta-analyses

Table 5 presents the results of time-to-event meta-analyses in included reviews. According to the confidence intervals of individual time-to-event meta-analyses, most reviews included at least one analysis which showed statistically significant or non-significant effects in favor of the intervention as indicated by the review authors. Most analyses showed relative risk reductions between 1 and 0.5 as indicated by their point estimates.

**Table 5:** Summary of time-to-event meta-analyses results in the included reviews

			Reviews			TTE outcomes	
		Overall (*N* = 100)	Cochrane (*n* = 50)	Non-Cochrane (*n* = 50)	Overall (*N* = 217)	Cochrane (*n* = 93)	Non-Cochrane (*n* = 124)
Result*	Favourable, statistically significant	52% (52)	40% (20)	64% (32)	40% (86)	32% (30)	45% (56)
	Favourable, statistically non-significant	57% (57)	54% (27)	60% (30)	38% (83)	40% (37)	37% (46)
	Unfavourable, statistically significant	7% (7)	6% (3)	8% (4)	5% (10)	3% (3)	6% (7)
	Unfavourable, statistically non-significant	25% (25)	36% (18)	14% (7)	16% (35)	23% (21)	11% (14)
	Direction of effect undetermined (HR = 1)	3% (3)	4% (2)	2% (1)	1% (3)	2% (2)	1% (1)
*Size of HR point estimate*	> 2	5% (5)	6% (3)	4% (2)	3% (6)	4% (4)	2% (2)
	< 2, >1	24% (24)	34% (17)	14% (7)	18% (38)	20% (19)	18% (19)
	1	3% (3)	4% (2)	2% (1)	1% (3)	2% (2)	1% (1)
	< 1, > 0.5	83% (83)	72% (36)	94% (47)	72% (156)	66% (61)	77% (95)
	< 0.5	13% (13)	12% (6)	14% (7)	6% (14)	8% (7)	6% (7)
*I* ^*2*^ *value*	Median (IQR)	NA	NA	NA	5 (0–48.81)	0 (0–41.935)	20 (0–55.9)
	Mean (range)	NA	NA	NA	25.36 (0–97)	21.22 (0–85.36)	28.55 (0–97)
	Not reported	NA	NA	NA	3% (6)	1% (1)	4% (5)
*HR for event or non event*	Event	98% (98)	98% (49)	98% (49)	99% (214)	99% (92)	98% (122)
	Unclear	2% (2)	2% (1)	2% (1)	1% (3)	1% (1)	2% (2)
*Interpretation of HR < 1* ^*#*^	Decreased risk of event	96% (96)	96% (48)	96% (48)	98% (212)	98% (91)	98% (121)
	Increased risk of event	2% (2)	2% (1)	2% (1)	1% (2)	1% (1)	1% (1)
	Unclear	2% (2)	2% (1)	2% (1)	1% (3)	1% (1)	2% (2)
*Trial HRs inverted*	Not reported	100% (100)	100% (50)	100% (50)	100% (217)	100% (93)	100% (124)

Abbreviations: HR = hazard ratio; IQR = interquartile range

* The designation “favourable/ unfavourable” is based on the review authors definition of the intervention, e.g., specified in Summary of Findings tables, e.g., “favourable”, when they interpreted a HR < 1 as beneficial for their designated intervention. It is based on the point estimate. ^#^ Interpretation of a HR < 1 as decreased/increased risk of the event is based on the review authors designated intervention and their interpretation. If in their forest plot a HR < 1 for overall survival was reported as “favors the intervention”, for example, it was clear that the HR represents a decreased the risk of the event (death), even though it is referred to as absence of the event (overall survival) by the review authors

Regarding the directionality of the HR as a relative effect measure, in almost all reviews the pooled HRs were applicable to events, meaning that review authors considered occurrence of the event of interest as basis for their calculation, and a pooled HR ≤ 1 indicated a decreased risk. Inversion of trial HRs in accordance with the direction of the meta-analysis HR was reported for none of the time-to-event outcomes.

### Handling of specific trial characteristics with relevance for time-to-event outcome analysis and interpretation

Heterogenous outcome definitions between trials were discussed in eight reviews (appendix-table A10, Additional File 1). Follow-up durations of included trials were frequently not specified. If they were, most often, reviews (5%, 5/100) reported a required minimum duration for all review outcomes or, for individual time-to-event outcomes, reported to use the longest follow-up available for each trial (8% (8/100 reviews)). Few reviews indicated how they dealt with potential varying follow-up between trials, e.g., through sensitivity analyses or meta-regression. Some mentioned varying follow-up times in the results and discussion sections.

Some form of handling of missing outcome data in trials was mentioned in all CR and 48% (24/50 reviews) of nCR. Most review authors included it among risk of bias criteria and it was mentioned in the majority of reviews, but outcome specifically only in two CR. Informative censoring, competing events, in particular deaths as competing events, and treatment switching were reported as risk of bias criteria and otherwise mentioned in the results and discussion sections of singular reviews. Three reviews described sensitivity analyses for treatment switching, e.g., according to the rates of switchers. No review reported an assessment of the proportional hazards assumption of included time-to-event analyses.

### Absolute effects for communication of findings

44% (44/100 reviews; CR: 80% (40/50 reviews), nCR: 8% (4/50 reviews)) of reviews used complementary absolute effect estimates (e.g., natural frequencies or risk differences) that were calculated based on the pooled HRs to communicate the results of their time-to-event meta-analyses (appendix-table A11, Additional File 1). None of the reviews reported the use of more complex and direct methods to estimate absolute effects for their meta-analyzed time-to-event outcomes, such as separate meta-analyses of the risk difference, simulation or bivariate models incorporating a baseline risk in the meta-analysis [29]. In six reviews, authors did explicitly not calculate absolute effects because outcomes “were time-to-event outcomes”. Most frequently used were natural frequencies, e.g., the number of participants experiencing an event until a given time-point among 1000 participants under observation. Baseline risks used to calculate absolute effects were mostly applicable to events. In a third of reviews, the outcome descriptions did not match the absolute effect descriptions (e.g., overall survival used for result reporting throughout the review, but absolute effects reported as all-cause mortality). The absolute effect description was adapted in seven CR with reasoning and in two reviews without any reasoning. Overall, correctly calculated and reported absolute effects were available in about a quarter (22% (22/100 reviews); CR: 40% (20/50 reviews), nCR: 4% (2/50 reviews)) of reviews. Correctly calculated but discrepantly labeled absolute effects were available in 10% (5/50 reviews) of CR. They were incorrect in 12% (6/50 reviews) of CR, where a HR for events was multiplied with a baseline risk for absence of events. In 7% (7/100 reviews) of reviews the interpretation of the absolute effect was unclear, because the direction of the baseline risk was not clear.

## Discussion

In summary, the majority of reviews that included meta-analyses of time-to-event outcomes addressed neoplasms and assessed all-cause mortality. Less than half of their analyzed outcomes were time-to event outcomes and outcome definitions for time-to-event outcomes were provided only in half of reviews.

The eligible analyses, e.g., intention-to-treat or per protocol, or unadjusted or adjusted analyses, were not always reported and information regarding the actually included analyses was rarely given. Methods to obtain time-to-event data varied substantially, prominently reported were direct inclusion of the HR and complete sets of recalculation methods, and were seldomly provided for individual outcomes.

In most cases the random-effects model was used for summarizing the estimated study treatment effects. The most prominently used method was the DerSimonian-Laird method [30]. Only in two reviews the Hartung-Knapp-Sidik-Jonkman (HKSJ) method was used, contrary to the recommendation of using HKSJ as standard approach for random-effects meta-analyses in the literature [31].

Although outcomes were most frequently presented in text as absence of event, the meta-analyzed HRs were almost entirely calculated based on rates of events and a HR < 1 indicated a lower risk in experimental arm. The results of the assessed meta-analyses were predominantly favorable (in 78%). Absolute effects based on these results were common in CR, however, in some reviews these were described or calculated incorrectly.

Lastly, trial characteristics with relevance to time-to-event outcome analysis, for example varying follow-up, adjusted/unadjusted effect estimates, informative censoring, competing events, treatment switching and proportional hazards, were only infrequently included in additional assessments (e.g., sensitivity analyses, risk of bias or certainty assessments) and seldomly mentioned throughout review texts at all.

To our knowledge, and in contrast to previous methodological surveys of general systematic review methods and reporting, this is the first assessment of time-to-event specific methods and time-to-event specific reporting of a sample of systematic reviews [27, 32]. For reporting of time-to-event analyses in study publications, several previous assessments found considerable limitations: e.g., infrequent reporting of start and end points of the observation, censoring reasons, follow-up times and calculation, as well as exact numbers of events and censored observations [13–17]. Furthermore, assumptions of the models used, such as the proportional hazards assumption, were often not verified or important details of statistical modelling were withheld [14, 16].

These findings were confirmed by a recent study that assessed trials included in meta-analyses of a random subset of the here analyzed reviews [33]. The study identified shortcomings in the reporting of time-to-event analyses in trial publications that are consistent with the limitations visible in review publications. These shortcomings relate to, for example, outcome definitions and trial characteristics relevant for the validity of time-to-event analyses. Some items, such as the types of analyses (e.g., intention-to-treat) and the adjustment of estimates were more consistently reported in trial publications than in their including reviews. Limitations and discrepancies in reporting of time-to-event analysis in trial publications introduce considerable difficulties for review authors. It seems reasonable to assume that the problematic reporting at trial level has an impact on reporting on review level. However, the limited reporting in trial publications was hardly or not at all addressed in the reviews themselves.

Methodological guidance for including time-to-event data in aggregate data meta-analyses exists and authors should follow these guidelines [1, 22, 28]. Irrespective of whether lack and variability of reporting in many of our assessed items translates into a neglect of the respective issues, limited reporting in review publications endangers misinterpretation of effects and certainty therein. For example, in a few cases the directionality of the pooled HR was unclear, either because it remained unclear whether the HR was representative of events or the absence of events, or because it was unclear whether an HR < 1 favored the intervention or the comparator. At worst, this can lead to a misinterpretation of effects in the opposite direction.

To enhance the quality of systematic reviews including aggregate data meta-analyses of time-to-event outcomes, a central implication of our assessment is the need for reporting standards that extend the currently available general reporting guidance [23]. While reporting standards for study publications that report on time-to-event analyses have been proposed, e.g., by Altman et al. [15] and Abraira et al. [14], such standards are currently not available for systematic reviews. As a product of the here reported methodological study, a respective reporting guideline is currently in development [34]. In addition, focused methodological guidance could increase review conductors’ awareness for more specific hardships, e.g., informative censoring, competing events and proportional hazards [5, 24]. Editors of biomedical journals also have a role in assuring the quality of the systematic reviews they publish. Without standards for the analysis and reporting of time-to-event outcomes in systematic reviews, they face certain difficulties. Future guidelines would therefore also be extremely helpful for them.

Variability in reported sources for time-to-event data in included reviews is a core finding of our study. Exclusion of studies because of perceived insufficient data does not only lead to less precise meta-analysis results but might also cause bias. The variety of approaches to recalculate time-to-event summary data from primary trials is therefore a critical predictor of review quality. In addition, the robustness of individual recalculation methods should be considered, and hierarchical selection of approaches could be advised. A recent study, for example, compared survival curve-based approaches to published HR and found that the method to reconstruct individual participant data by Guyot et al. [35] had the smallest bias and largest precision. Should established recalculation methods fail due to absence of data, it must be discussed whether to resort to less conventional methods. In an assessment of Cochrane reviews, Salika et al. [18] found that binary analysis of time-to-event outcomes could be feasible under low event probabilities. They suggest complementary log-log links as possible option for the case that only binary data are available. Particularly for situations with non-proportional hazards and for ease of interpretation, meta-analysis on the restricted mean survival time is an attractive option, which requires, however, recalculation of individual participant data using one of many available approaches [36].

### Strengths and limitations

We provide an in-depth view on the current landscape of systematic reviews following rigorous, prespecified methods. Duplicate performance of relevant steps and structured forms warrant robustness despite the considerable extent of data. To deal with the large amount of data for extraction, we chose a two stage process: First, we extracted the data for 25 reviews in duplicate and then, with sufficient agreement (less than < 10% discrepancy), we continued extracting data in a double-checked manner, with one project participant performing the extraction and a second independent project participant checking the extracted data in the review publications.

We aligned our sample on a fixed number of CR to ensure a view on methods and reporting in reviews from a well-established source often considered the gold-standard. This may have led us to underestimate the size of the observed problems in the overall biomedical literature. For inference to the general landscape of systematic reviews, we included a large number of non-Cochrane reviews. We restricted the reviews to those published in Core Clinical Journals to ensure an elevated publication standard of the reviews, thereby also endangering possible underestimation of visible problems in relation to the overall literature. Because we identified a larger number of potentially eligible non-Cochrane reviews and for feasibility of our endeavor under its set goals, we drew a random sample from the non-Cochrane reviews stratified by publication years to meet the number of Cochrane reviews. To limit any potential selection bias, despite the application of the Core Clinical Journals filter, the random selection was performed independently by a project participant not involved in the selection of articles and extraction of data.

We limited our assessment to comparisons of ≤ 20 trials for feasibility of a separate, independent analysis step, which could impact the results [33]. As the total number of excluded reviews was minimal, 3% CR and 5% nCR during full-text screening, we believe the impact to be minimal. Finally, and relevant for literature-based methodological assessments in general, we must stress that review reporting does not necessarily correspond with review performance, e.g., regarding authors approaches to retrieve time-to-event data. Yet, reporting of analytic steps may reflect their relative importance to authors and might constitute a sufficient surrogate for their performance.

## Conclusions

We identified variable and often insufficient reporting of time-to-event specific features in current systematic reviews including aggregate data meta-analyses based on the HR. Review authors should rigorously utilize available methodological guidance for the conduct of their analysis. Reporting standards might improve the quality of respective reviews.

## Electronic supplementary material

Below is the link to the electronic supplementary material.

Supplementary Material 1: Search strategy and extending tables

## Data Availability

The data supporting the conclusions of this article are presented in the manuscript and in extending tables in the appendix (Additional File 1). All raw data is available as Excel file upon request.

## Declarations

### Registration

This meta-epidemiological study is registered under osf.io/5qxbd.

## Abbreviations

ACM: All-cause mortality
CR: Cochrane review
GRADE: Grading of Recommendations Assessment, Development and Evaluation
HKSJ: Hartung-Knapp-Sidik-Jonkman
HR: hazard ratio
IPD: Individual participant data
IQR: Interquartile range
MA: Meta-analysis
nCR: Non-Cochrane review
OS: Overall survival
PRISMA: Preferred Reporting Items for Systematic Reviews and Meta-Analyses
RCT: Randomized controlled trial
TTE: Time to event
