# Supplementary material for: Exploring the characteristics, methods and reporting of systematic reviews with meta-analyses of time-to-event outcomes: a meta-epidemiological study
Source: BMC Med Res Methodol. 2024 Nov 25;24:291. doi: 10.1186/s12874-024-02401-4 (PMC11587663; doi:10.1186/s12874-024-02401-4)
Supplement: Supplementary file 1 — Supplementary Material 1: Search strategy and extending tables [file 12874_2024_2401_MOESM1_ESM.docx]

# Appendix

## Appendix A1: Complete search strategy for non-Cochrane reviews

Medline on February 8^th^, 2021

# Searches

1 "time-to-event".tw,kf.

2 "log rank".tw,kf.

3 survival.tw,kf.

4 hazard.tw,kf.

5 Kaplan-meier estimate/

6 kaplan-meier.tw,kf.

7 (method* adj1 (product* or limit*)).tw,kf.

8 (cumulative* adj1 incidence*).tw,kf.

9 outcome expectation.tw,kf.

10 (cox adj2 (model* or proportional*)).tw,kf.

11 proportional hazards models/

12 or/1-11

13 (randomi?ed or placebo or randomly).ab.

14 meta analysis.mp,pt.

15 12 and 13 and 14

16 limit 15 to dt=20170101-20200801

## Appendix A2: List of extraction items

***Extraction items for reviews***

| # | Feld | Options | Description |
| --- | --- | --- | --- |
| 1 | ID of the assessed reviews |  | Individual number of review |
| 2 | Trial level extraction? | Yes; No |  |
| 3 | Type of assessed review | Cochrane review; Non-Cochrane review |  |
| 4 | Last name of first author |  |  |
| 5 | Publication year |  |  |
| 6 | If non-Cochrane review: Journal? |  | Please insert the full name of the journal that published the assessed review |
| 7 | Review update? |  | Is this a review update? |
| 8 | Medical field | Infections and parasitic diseases;  Neoplasms (oncological studies irrespective of the field are sorted into this category);  Diseases of the blood, blood-forming organs and the immune mechanism;  Endocrine, nutritional and metabolic diseases;  Mental and behavioral disorders;  Diseases of the nervous system;  Diseases of the eye and adnexa;  Diseases of the ear and mastoid process;  Diseases of the circulatory system;  Diseases of the respiratory system;  Diseases of the digestive system;  Diseases of the skin and subcutaneous tissue;  Diseases of the musculoskeletal system and connective tissue;  Diseases of the genitourinary system;  Pregnancy, childbirth and the puerperium |  |
| 9 | Multiple comparisons? | Yes; No |  |
| 10 | Comments on review information | Comments on general review information |  |
| 11 | Medical condition |  | Medical condition of the assessed population  e.g., Melanoma, 1st line, NSCLC, Squamous, 1st line, Brain Tumor WHO Grading II, 1st line  "Not specified": if not particular stage of disease is defined, an eligibility criterion or otherwise identifiable |
| 12 | Clinical stage |  | Clinical stage of condition of assessed population  E.g., primary occurrence of disease, primary recurrence of disease, multiple recurrences of disease, chronic disease etc. |
| 13 | What was the assessed age group? | Adults; Pediatric; Both; Not reported | Adults if participants included with upper age limit in adult population (e.g., ≥18 years, ≥16 years) Pediatric if limited to youth population (e.g., <18 years) Both if explicitly not age limit applied  Interpretation: „Adults“ = Adults or not otherwise reported (besides explicit reporting, age in included trials and relevance of disease in population feasible criteria for judgement) |
| 14 | Experimental intervention | Biologics/ drug; Surgical procedure; Medical devices; Behavioral intervention; Exercise intervention; Screening; Radiation; Absence of intervention; Other (please specify) | If possible, choose experimental intervention reported in first Summary of Findings table, otherwise use experimental intervention specified in abstract |
| 15 | Specify experimental intervention |  |  |
| 16 | Control intervention | Placebo; No treatment; Usual care; Biologics/ drugs; Surgical procedure; Medical devices; Behavioral intervention; Exercise intervention; Screening; Radiation; Other (please specify); Best supportive care/ Optimal medical care; Observation | If possible, choose control intervention reported in first Summary of Findings table, otherwise use control intervention specified in abstract |
| 17 | Specify control intervention |  |  |
| 18 | Comparator treatment considered? | Yes (Control group received intervention); Yes (Experimental group received control); Yes (Both possible); No | Reception of a comparator treatment in either intervention group explicitly considered? |
| 19 | Planned outcome number |  | Number of planned outcomes  "Not clear" = if counting of outcomes not possible "Not reported" = no planned outcomes reported  e.g., overall survival, progression-free survival, ...  If "adverse events" with no further specification, please consider it as a single outcome, otherwise, count specification together with "Adverse events: ..., Adverse events: ..." |
| 20 | Planned TTE outcome number |  | Number of planned time-to-event outcomes  "Not clear" = if counting of outcomes not possible "Not reported" = no planned outcomes reported  e.g., overall survival, progression-free survival, ...  If "adverse events" with no further specification, please consider it as a single outcome, otherwise, count specification together with "Adverse events: ..., Adverse events: ..." |
| 21 | List of planned TTE outcomes |  | Please list the names of all outcomes that were to be assessed in the review as time-to-event outcomes  Names only, no definition, e.g., overall survival, progression free survival; Outcomes initially planned to be assessed as time-to-event but analyzed as binary should here be counted as TTE |
| 22 | Planned follow-up of review |  | Specify time-frame if it was mentioned (including exact durations and minimum durations; please copy and paste a respective sentence or describe appropriately)  Enter "Not reported" if no time-frame or any specification for follow-up duration was mentioned  Enter "Unclear" otherwise |
| 23 | Comments on PICO or time-frame |  | Comments regarding the review PICO or time-frame |
| 24 | Studies in quantitative analysis |  | Number of studies included in quantitative analysis  If number included reported without specification use this number   If number included in "qualitative synthesis" and "quantitative synthesis"/ meta-analysis are reported separately, please choose the number included in "quantitative synthesis"/ meta-analysis  If necessary type: "Unclear" "Not reported" |
| 25 | Total population in review |  | Size (number) of the total population included in quantitative analysis?  If number included reported without specification use this number   If number included in "qualitative synthesis" and "quantitative synthesis"/ meta-analysis are reported separately, please choose the number included in "quantitative synthesis"/ meta-analysis  If necessary type: "Unclear" "Not reported" |
| 26 | Experimental population in review |  | Size (number) of the experimental population included in the review?  Only if number explicitly reported (number of participants in arms from forest plot relevant on review outcome level)  If number included reported without specification use this number   If number included in "qualitative synthesis" and "quantitative synthesis"/ meta-analysis are reported separately, please choose the number included in "quantitative synthesis"/ meta-analysis  If necessary type: "Unclear" "Not reported" |
| 27 | Control population in review |  | Size (number) of the control population included in the review?  Only if number explicitly reported (number of participants in arms from forest plot relevant on review outcome level)  If number included reported without specification use this number   If number included in "qualitative synthesis" and "quantitative synthesis"/ meta-analysis are reported separately, please choose the number included in "quantitative synthesis"/ meta-analysis  If necessary type: "Unclear" "Not reported" |
| 28 | Number of outcomes analyzed |  | Number of all outcomes analyzed  Number of main meta-analyses, disregard narrative, sensitivity and subgroup analyses  If necessary type: "Unclear" "Not reported" |
| 29 | Number of TTE outcomes analyzed |  | Number of outcomes analyzed as TTE outcomes  Number of main meta-analyses only, disregard narrative, sensitivity and subgroup analyses; Outcomes that were initially planned to be assessed as TTE outcomes but analyzed as binary outcomes (e.g., due to data issues) in the review should NOT be counted here.  If necessary type: "Unclear" "Not reported" |
| 30 | Comments regarding the sample size |  |  |
| 31 | HR / log(HR) in analysis | HR/ log(HR) not further specified; HR/ log(HR) (Cox model); HR/ log(HR) (log-rank); HR/ log(HR) (parametric model); HR/ log(HR) (Calculated from Kaplan Meier); other (specify); unclear; not reported | What types of hazard ratios/ log(HR)s did the review authors plan to include in their time-to-event meta-analyses? |
| 32 | Methods to obtain TTE data | HR and confidence intervals; HR together with other information (e.g., events in each arm, total events, etc.); O & E or hazard rates on research and control arm; O-E together with logrank V; P-value together with additional information (e.g., events, total events, etc.); Survival curves; Specified particular set of methods (e.g., Tierney 2008, Cochrane Handbook, etc.); Unclear; Not reported; Other (specify); log(HR) and standard error; Median survival times; Time point specific survival times; IPD (recalculated or from publication) | Methods the review authors specified to obtain time-to-event data from trials to pool time-to-event outcomes |
| 33 | Comment comparative effect measures |  | Please make a comment if the authors specified other methods to calculate HRs (e.g., parametric models) |
| 34 | Types of analysis for meta-analyses | ITT; mITT; PP; As treated; Unclear; Not reported | Types of analysis preferred to be included in main meta-analyses of review  mITT = "justified" exclusion of participants post randomization (e.g., ineligible participants; exclusion before reception of study treatment   Explicit preference or inclusion of presence/ absence of ITT in RoB assessments analyses can also give a hint on the target analysis of authors |
| 35 | Types of analyses in meta-analyses | ITT; mITT; PP; As treated; Unclear; Not reported; Other (specify); Not reported for all trials; Included trial(s) did not report type of analysis | Was it explicitly reported which analyses from trials were included in meta-analyses of this review?  Assessment on review level - if there is any specification of included analyses on review outcome level, please mark this item as „not reported“ and use respective field on review outcome level   It should be clear and explicitly reported which type of analysis was included from which trial (analyses reported only for individual trials are counted as "not reported for all trials" |
| 36 | Adjusted, unadjusted or both types of effects in meta-analyses | Adjusted only; Unadjusted only; Hierarchical (unadjusted before adjusted); Hierarchical (adjusted before unadjusted); Both; Unclear; Not reported | Did the review authors specify to include adjusted, unadjusted and/or both types of effects in the main meta-analyses of this review? |
| 37 | Handling of adjusted and unadjusted HR in meta-analysis | Combined in meta-analysis; Sensitivity analysis (two separate analyses); Other (please specify); Mentioned as RoB criterion in methods; Not applicable (unadjusted/ adjusted not mentioned); Unclear; Not reported | How did the authors plan to handle adjusted and unadjusted HRs in meta-analyses of this review? |
| 38 | Stratified effects eligible? | Yes; No; Unclear; Not reported | Please indicate whether stratified effect measures (e.g., stratified HRs) were eligible for meta-analysis?  Please choose yes only, if stratified effect measures where explicitly mentioned. Stratification can be seen as a form of adjustment. Not to be confused with randomization stratification or subgroup-analyses (analysis-results per stratum). |
| 39 | Comments on handling of adjusted and unadjusted hazard ratios | |  |
| 40 | Methods to pool TTE data | Inverse variance; Peto (fixed-effects) model; Other (specify); Unclear; Not reported | Which methods were planned to pool time-to-event data? (irrespective whether reported in forest plot; data in forest plots is relevant for the assessment on review outcome level) |
| 41 | Handling of heterogeneity | Random-effects meta-analysis performed; Subgroup analyses; No pooling if too heterogeneous; Other; Unclear; Not reported | How did the authors intent to handle heterogeneity between studies?  (Refers to planned handling of heterogeneity) |
| 42 | TTE meta-analysis models | fixed effects only; random effects only; mixed (either one as sensitivity analysis); fixed; and if not possible random effects; unclear; not reported | Model type for time-to-event meta-analyses |
| 43 | Other pooled TTE outcome measures | none; median survival time; restricted mean survival time; rank preserving structural failure; other (please specify); relative risk | Pooled analysis with other outcome measures besides HR for time-to-event outcomes?  e.g., pooling of median survival times; standard error of the combined log(MST) |
| 44 | Comments on meta-analytic methods |  |  |
| 45 | Dealing with varying follow-up | Sensitivity analyses (e.g., studies with shorter/longer follow-up time in separate analysis); Exclusion of studies with divergent follow-up time; Mentioned as RoB criterion in methods; Other (please specify); Not applicable, pre-defined timing as inclusion criterion; Unclear; Not reported | How did the authors intent to deal with varying follow-up times between the included trials? |
| 46 | Comments on treating follow-up times |  | Comments regarding the treatment of variable follow-up times |
| 47 | Handling of competing events | Subgroup analysis (e.g., according to competing event rate); Mentioned as RoB criterion in methods; Exclusion of trials above a certain rate of competing events; Other (please specify); Not applicable, no outcomes with potential competing events; Unclear; Not reported | How did the review authors plan to deal with competing events in time-to-event analyses of included trials? |
| 48 | Comments on treating competing events |  | Comments regarding the treatment of competing events in the meta-analysis for this outcome |
| 49 | Handling of MOD | Recalculation where possible; Single imputation; Multiple imputation; Meta-regression; Sensitivity analyses (according to rate of missing values); Mentioned as RoB criterion in methods; Contact with authors; Other (please specify); Unclear; Not reported | How did the authors intent to deal with missing outcome data in the included trials |
| 50 | Comments on the treatment of missing outcome data |  | Comments on the treatment of missing outcome data |
| 51 | Handling of non-administrative censoring | Sensitivity analysis (e.g., according to rate of censoring); Exclusion of trials (e.g., according to rate of censoring); Single imputation; Multiple imputation; Meta-regression; Mentioned as RoB criterion in methods; Other (please specify); Unclear; Not reported | How did the review authors intend to deal with censoring for non-administrative reasons (informative censoring) in the time-to-event analyses of the included trials?  Example for administrative reasons: end-of-study censoring |
| 52 | Comments on treating non-administrative censoring |  | Comments on treating non-administrative censoring |
| 53 | Handling of comparator treatments in participants | Complies with review PICO; No handling mentioned, probably ITT; Intention-to-treat; Per protocol; As treated; Sensitivity analysis (e.g., According to rate of participants); Mentioned as RoB criterion in methods; Other (please specify); Unclear; Not reported | How did the review authors intend to deal with the reception of comparator treatments in trial participants? |
| 54 | Comments on dealing with participants receiving comparator treatments | | Comments regarding the treatment of trial participants receiving comparator treatments |
| 55 | Proportional hazards assessment | Recalculation of IPD; Use of trial level tests; Use of other trial level data (e.g., visual inspection of survival curves); Both: trial level tests, if not provided inspection of curve; Unclear; Not reported; | How did the authors intend to assess the proportionality of hazards in included trials? |
| 56 | Handling non-proportional hazards | No action, all trials included regardless of prop. hazard assumption; Sensitivity analysis (e.g., according to degree of non-proportionality); Other (please specify); Not applicable (No assessment of proportionality reported); Unclear; Not reported | How did the authors intend to deal with non-proportional hazards in included trials? |
| 57 | Comments on proportionality of hazards |  | Comments on proportionality of hazards |
| 58 | RoB tool | no RoB assessment; RoB 1, study level; RoB 1, outcome level; RoB 2; Other | Which tool did the authors intend to use to assess bias in included trials? |
| 59 | TTE specific RoB assessment? | Yes; No; Unclear; Not applicable (no RoB assessment) | Did the authors intend to assess TTE specific trial characteristics in their RoB assessment? |
| 60 | If yes or unclear, please specify (RoB) |  | If yes or unclear, please specify (RoB) |
| 61 | Use of GRADE | Yes; No |  |
| 62 | TTE specific GRADE assessment? | Yes; No; Unclear; Not applicable (no GRADE assessment) | Did the authors intend to include time-to-event specific aspects in their GRADE rating? |
| 63 | If yes or unclear, please specify (GRADE) |  | If yes or unclear, please specify (GRADE) |
| 64 | Summary of findings tables for TTE outcomes? | Yes; No |  |
| 65 | Comments on risk of bias and GRADE |  | Comments on risk of bias and GRADE |
| 66 | Competing events discussed? | In results; In discussion; Not reported; Not applicable | Did the authors discuss competing events and/or their potential impact on review results? |
| 67 | Heterogenous outcome definitions discussed? | In results; In discussion; Not reported; Not applicable | Did the authors discuss heterogeneity of outcome definitions among included studies and/or its potential impact on review results? |
| 68 | Difference in follow-up times of trials discussed? | In results; In discussion; Not reported; Not applicable | Did the authors discuss differential trial follow-up for outcomes and/or their potential impact on review results? |
| 69 | MOD discussed? | In results; In discussion; Not reported; Not applicable | Did the authors discuss missing outcome data in included trials and/or its potential impact on review results? |
| 70 | Non-administrative censoring discussed? | In results; In discussion; Not reported; Not applicable | Did the authors discuss censoring for non-administrative reasons (informative censoring) and/or its potential impact on review results? |
| 71 | Reception of comparator treatments discussed? | In results; In discussion; Not reported; Not applicable | Did the authors discuss the reception of comparator treatments in trial participants and/or its potential impact on review results? |
| 72 | Adjusted or unadjusted estimates discussed? | In results; In discussion; Not reported; Not applicable | Did the authors discuss the inclusion of adjusted and unadjusted estimates for the same outcomes and/or its potential impact on review results? |
| Abbreviations: HR = hazard ratio, ITT, = intention to treat, mITT = modified intention to treat, MOD = missing outcome data, MST = mean survival time, PICO = population-intervention-comparator-outcomes, RoB = risk of bias | | | |

***Extraction items for individual review time-to-event outcomes***

| # | Item | Options | Description |
| --- | --- | --- | --- |
| 1 | Review ID |  | Review ID (number) assessable from Excel |
| 2 | Review outcome |  | Outcome ID (number) assessable from Excel  Please use review description (without definition) |
| 3 | Primary outcome? | Yes; No; Not applicable (No primary/ secondary outcome defined) | Was this outcome specified as a primary outcome of the review? |
| 4 | Complete outcome definition |  | Please provide the complete definition of the outcome |
| 5 | Composite outcome | Yes; No; Unclear; Not reported | Does this outcome include multiple outcome events?   for example progression free-survival - progression, relapse and death from any cause |
| 6 | Composite events described? | Yes; No; Not applicable ("composite outcome" unclear or not reported) | Were the outcome events composing this composite outcome described? |
| 7 | All-cause mortality part of outcome? | Yes; No; Unclear | Was all-cause mortality a component of the assessed outcome? |
| 8 | Competing events possible? | Yes; No; Unclear | Are competing events possible by definition of the outcome?  Choose yes, e.g., if overall mortality was not part of the defined outcome |
| 9 | Reported as event or absence of event? | Event; Absence of event; Both (with reasoning); Unclear (both without reasoning, e.g., switching of reporting) | Was the outcome reported as event (e.g., death, relapse) or absence of event (e.g., overall survival, progression-free survival)?  Refers to the description of the outcome overall (e.g., in the methods section, headlines, etc.)  (Presentation of the pooled results is assessed in a subsequent item) |
| 10 | Follow-up pre-specified? | Time-specific (12 months, 2 year, 10 year, ...); Longest follow-up; Minimum duration of follow-up required; Maximum duration of follow-up specified; Not reported | Was a duration of follow-up included in definition or otherwise pre-specified for the outcome? |
| 11 | Start of outcome assessment defined | Yes; No; Not applicable (e.g., outcome not defined); Unclear | Was the start of outcome assessment for this outcome included in the outcome definition or otherwise prespecified in the statistical methods section? |
| 12 | Outcome assessment start | Randomization; Enrollment; Allocated treatment; Previous treatment (e.g., surgery); Other (specify); Not applicable (e.g., start of follow-up not reported); Unclear | What was the defined or otherwise reported start time-point of outcome assessment for this outcome?  MG: Nachträglich eingefügt: Für bereits extrahierte: Wenn auf Basis der extrahieren Definitionen nicht nachverfolgbar bitte offen lassen (ich sollte es extrahiert haben) |
| 13 | Field for commenting on outcome |  | Field for commenting on the outcome (e.g., what was unclear, whether name changes occurred during the reviews (OS to All cause mortality), other irregularities, etc.) |
| 14 | Types of analyses ELIGIBLE for meta-analysis | ITT; mITT; PP; Other (please specify); Unclear; Not reported | Please indicate what types of analyses (e.g., ITT, PP) were ELIGIBLE in the assessed meta-analysis |
| 15 | Types of analyses INCLUDED in meta-analysis | ITT; mITT; PP; Other (please specify); Unclear; Not reported | Please indicate what types of analyses (e.g., ITT, PP) were INCLUDED in the assessed meta-analysis (as reported by the review authors) |
| 16 | Comments on eligible or included analyses |  |  |
| 17 | Varying follow-up times handling specifically reported | Yes; No | Was handling of varying follow-up specifically reported for this outcome? |
| 18 | Handling of varying follow-up times | Sensitivity analysis (studies with shorter/ longer follow-up time in separate analysis); Exclusion of studies with divergent follow-up time; Other (please specify); Pre-defined timing as inclusion criterion; Not applicable | Please indicate whether and how the authors dealt with varying follow-up times between the included trials for this meta-analysis |
| 19 | Comments on variable follow-up times in trials included in meta-analysis |  |  |
| 20 | Competing events handling specifically reported | Yes; No | Was handling of competing events specifically reported for this outcome?  ("No" also when competing events not possible) |
| 21 | Handling of competing events | Sensitivity analysis (e.g., according to competing event rate); Exclusion of studies above a certain rate of competing events; Other (please specify); No outcome with potential competing events | Please indicate how the authors reported to or treated competing events in the meta-analysis of this outcome |
| 22 | Commenting on competing events in the meta-analysis |  | Comments on competing events in the meta-analysis of this outcome |
| 23 | Missing data handling specifically reported | Yes; No | Was handling of missing data specifically reported for this outcome |
| 24 | Handling of MOD | Recalculation where possible; Contact with authors; Single imputation; Multiple imputation; Meta-regression; Sensitivity analyses (according to rate of missing values); Hierarchy of the above (please specify); Other or multiple (please specify) | How did the authors treat missing outcome data in the meta-analysis of this outcome? |
| 25 | Comments on MOD in the meta-analysis |  | Comments on missing outcome data in the meta-analysis of this outcome |
| 26 | Censoring handling specifically reported | Yes; No | Was handling of censoring for non-administrative reasons reported specifically for this outcome?  "Censoring" should explicitly be mentioned for a judgement |
| 27 | Treatment of censoring for non-administrative reasons | Sensitivity analysis (e.g., according to rate of censoring); Exclusion of trials (e.g., according to rate of censoring); Single imputation; Multiple imputation; Meta-regression; Other or multiple (please specify) | Please indicate how the authors treated censoring of participants for non-administrative reasons (informative censoring) in the meta-analysis of this outcome |
| 28 | Comments on treatment of non-administrative censoring |  | Comments on the treatment of non-administrative (informative) censoring in the meta-analysis of this outcome |
| 29 | Comparator treatments handling specifically reported | Yes; No | Was handling the reception of comparator treatments specifically reported for this outcome? |
| 30 | Treatment of comparator treatments | Complies with review PICO; No handling mentioned, ITT; Per protocol analysis; Sensitivity analysis (e.g., According to rate of participants); Other or multiple (please specify) | How did the authors treat the reception of comparator treatments in trial participants in the meta-analysis of this outcome |
| 31 | Comments on comparator treatments in meta-analysis |  | Comments on the treatment of trial participants receiving comparator treatments in the meta-analysis for this outcome |
| 32 | Absolute effect measures reported? | Yes; No; Absolute effects explicitly not calculated ("e.g., not calculable because of TTE outcome") | Where absolute effect measures based on the outcomes of this meta-analysis calculated and provided? |
| 33 | Type of absolute effects | Natural frequencies; Risk difference; NNT; Median survival or difference in median survival; Not applicable | What type of absolute effects where calculated and reported? |
| 34 | Baseline risk applicable for events or absence of events | Event; Absence of event; Unclear; Not applicable | If a baseline risk (e.g., control group risk) was used for the calculation of absolute effects (e.g., in a SoF table), was it applicable for events or absence of events  Event (e.g., death (mortality), relapse, etc.): Number of individuals with composite outcome (e.g., "relapse or death" (falsely: PFS)) HIGHER than single event outcome (e.g., "mortality" (falsely: OS)) (when composite is including the single event outcome) (e.g., "Relapse or death"/ PFS: 200 vs. "Mortality"/OS: 100)  Absence of event (e.g., OS, PFS, EFS): Number of individuals with composite outcome (e.g., PFS (falsely: "relapse or death")) LOWER than single event outcome (e.g., OS (falsely: "mortality")) (when composite is including the single event outcome) (e.g., "Relapse or death"/ PFS: 800 vs. "Mortality"/OS: 900)  Unclear: No reported or conflicting information which type of baseline risk was chosen (e.g., baseline risks from multiple sources so that the above rule does not apply, same baseline risk for different outcomes (e.g., OS=PFS), no comparison possible) |
| 35 | Description of outcome event or direction changed for calculation | No (no changes); Yes (description changed with reasoning); Yes (description changed without reasoning); Yes (HR inverted, with reasoning); Yes (HR inverted, without reasoning); Unclear; Not applicable | Was the description of the type of outcome event (absence of event to event (overall survival to all cause mortality) or the direction of the effect estimator (e.g., by inversion) changed for the calculation of absolute effects? |
| 36 | Absolute effects correct? | Yes; No; Correct calculation but wrong labeling; Unclear (e.g., not clear what the baseline risk is applicable for or unclear whether the HR corresponds to absence of events or events); Not applicable | Were absolute effects calculated correctly for this outcome?  (false labelling refers to e.g., mortality (event) labelled as overall survival (event-free))  Please check the SoF table first |
| 37 | Absolute effects comment |  |  |
| 38 | HR type specifically reported | Yes; No | Were the type or characteristics of the summarized hazard ratio (e.g., Cox, parametric model, log-rank, etc.) specifically described for this outcome? |
| 39 | Summarized HR | HR/ log(HR) not specified; HR/ log(HR) (Cox model); HR/ log(HR) (log-rank); HR/ log(HR) (parametric model); HR/ log(HR) (Calculated from Kaplan Meier); Other (specify); Unclear; Not reported | Type of HR summarized in this analysis? |
| 40 | Comments on HR |  | Comments on the hazard ratio that was included in the meta-analysis |
| 41 | Recalculation of HRs specifically reported | Yes; No | Did the authors report on whether and/or how they recalculated HRs specifically for this outcome?  Choose "no" of only review level or no information was reported |
| 42 | Retrieval of TTE data | HR and confidence intervals; HR together with other information (e.g., events in each arm, total events, etc.); O & E or hazard rates on research and control arm; O-E together with logrank V; P-value together with additional information (e.g., events, total events, etc.); Survival curves; Other (specify); Not specified for this outcome; Unclear; Median survival times; Time-point specific survival times; IPD (recalculated or from publication); Reported, but method not clear | How did the authors obtain time-to-event data to pool for this particular outcome? |
| 43 | Method to pool TTE data | Inverse variance method; Peto (fixed-effects) method; HKSJ (random-effects); Other (specify); Unclear; Not reported | Which method was used to pool time-to-event data in this meta-analysis? |
| 44 | Model used for meta-analysis | Fixed effects; Random effects; Both, one as sensitivity analysis; Unclear; Not reported | Which model was used for this meta-analysis? |
| 45 | Heterogeneity handling specifically reported | Yes; No | Did the authors report on how they dealt with heterogeneity between studies specifically for this outcome  Choose "no" of only review level or no information was reported |
| 46 | Dealing with heterogeneity | Random-effects MA; Subgroup analyses; Other (specify); Unclear; No heterogeneity | How did the authors deal with heterogeneity between studies in the performed meta-analysis? |
| 47 | Comment on handling heterogeneity |  | Comments on the handling of heterogeneity for this particular outcome |
| 48 | Proportional hazard handling specifically reported | Yes; No | Did the authors specifically report dealing with proportional hazards for this outcome?  Choose "no" of only review level or no information was reported |
| 49 | Test for proportionality of hazards | Recalculation of IPD; Use of trial level tests; Use of other trial level data (e.g., visual inspection of survival curves); Both: trial level tests, if not provided inspection of curve; No test of proportional hazards done; Unclear | Please indicate whether and through which test the review authors assessed the proportionality of hazards in trials included in this meta-analysis |
| 50 | Non-proportionality of hazards indicated by tests? | Yes; No; Not applicable; Unclear | Did the test for proportionality that review authors performed indicate a problem with non-proportionality of hazards?  (According to the review authors) |
| 51 | Dealing with (non-)proportionality of hazards | No action, all studies included regardless of prop. hazard assumption; Sensitivity analysis (e.g., According to degree of non-proportionality); Other (please specify); Unclear; Not reported; Not applicable | Please indicate whether and how the review authors dealt with the (non-)proportionality of hazards in the trials included in this meta-analysis |
| 52 | Comment on proportionality of hazards in trials included in this meta-analysis |  |  |
| 53 | Adjusted and unadjusted HRs specifically reported | Yes; No | Did the authors report on the inclusion of adjusted and unadjusted hazard ratios specifically for this outcome?  Choose "no" of only review level or no information was reported |
| 54 | Adjusted or unadjusted HRs included? | Adjusted only; Unadjusted only; Hierarchical selection (unadjusted before adjusted); Hierarchical (adjusted before unadjusted); Both; Unclear | Were adjusted or unadjusted hazard ratios included in this meta-analysis? |
| 55 | Dealing with with adjusted and unadjusted HR | Combined in same meta-analysis; Sensitivity analysis (two separate analyses); Other (please specify); Not applicable | Please indicate how the review authors dealt with adjusted and unadjusted hazard ratios in the conducted meta-analyses |
| 56 | Comments on handling adjusted and unadjusted HRs |  | Comments on the treatment of adjusted and unadjusted hazard ratios in this meta-analysis |
| 57 | Number of studies |  | Number of studies included in this meta-analysis  If not directly reported (e.g., in SoF), please recalculate from forest plot |
| 58 | Participants in experimental arm |  | Total number of participants in experimental arm of the assessed meta-analysis If not directly reported (e.g., in SoF), please recalculate from forest plot Empty field = "Not reported/ Unclear" |
| 59 | Participants in control arm |  | Total number of participants in control arm of the assessed meta-analysis  If not directly reported (e.g., in SoF), please recalculate from forest plot Empty field = "Not reported/ Unclear" |
| 60 | Total participants |  | Total number of participants in control arm of the assessed meta-analysis  Empty field = "Not reported/ Unclear" |
| 61 | Comments on sample size and number of studies |  |  |
| 62 | Pooled HR |  | What was the pooled hazard ratio for this outcome? |
| 63 | Lower 95% CI |  | What was the lower 95% CI of the HR for this outcome? |
| 64 | Upper 95% CI |  | What was the upper 95% CI of the HR for this outcome? |
| 65 | Chi² |  | What was the value of the Chi² statistic in the meta-analysis for this outcome?  Empty field = "Not reported/ Unclear" |
| 66 | I² |  | What was the value of the I² statistic for this outcome?  Empty field = "Not reported/ Unclear" |
| 67 | HR for events or absence of events | Event; Absence of event; Inconsistently (e.g., "OS" in abstract or meta-analysis and "risk of death"/ "mortality" in results for the same HR); Reasonable variation (e.g., "mortality" in results and inverted to "OS" in SoF); Unclear | Was the pooled hazard ratio reported as applicable for events or for absence of events (in the abstract, results section and (where applicable) SoF)  Negative events (e.g., death), when HR <1 reported as beneficial Absence of negative event (e.g., OS, PFS) when HR >1 reported as beneficial  Positive event when HR >1 reported as beneficial Absence of positive event when >1 HR reported as beneficial |
| 68 | HR <1 an increased or decreased risk of event in experimental group | Increased risk; Decreased risk; Unclear | Does a hazard ratio <1 indicate an increased or decreased risk of the event in the group that is assessed as experimental group in the review publication? |
| 69 | HRs inverted? | Yes; Unclear; Not reported | Did the authors describe that hazard ratios from trial publications were inverted to correspond to the direction of the hazard ratio in this meta-analysis? |
| 70 | Comments on meta-analysis results |  | Field for comments on the results of the meta-analysis |
| 71 | Outcome in SoF | Yes; No; Not applicable | Was the assessed outcome presented in a summary of findings table? |
| 72 | Overall GRADE rating | High; Moderate; Low; Very low; Not applicable | What was the overall GRADE rating for the assessed outcome? |
| 73 | Study limitations rating | 0; 0.5; 1; 2; Not applicable | Did the review authors rate down for study limitations? |
| 74 | TTE-specific study limitations | Yes (specify); No; Unclear; Not applicable | Did the review authors considerer TTE-specific study limitations in their GRADE assessment?  "Not applicable" = No GRADE rating or not downgraded for this particular domain  (e.g., naive inclusion of competing events or informative censoring) |
| 75 | Imprecision rating | 0; 0.5; 1; 2; Not applicable | Did the review authors rate down for imprecision in their GRADE assessment? |
| 76 | TTE-specific imprecision | Yes (specify); No; Unclear; Not applicable | Did the review authors consider TTE-specific sources of imprecision in their GRADE assessment?  "Not applicable" = No GRADE rating or not downgraded for this particular domain   (e.g., too short follow-up period; high rates of censoring; low number of overall events) |
| 77 | Indirectness rating | 0; 0.5; 1; 2; Not applicable | Did the review authors rate down for indirectness? |
| 78 | TTE-specific indirectness | Yes (specify); No; Unclear; Not applicable | Did the review authors consider TTE-specific sources of indirectness?  "Not applicable" = No GRADE rating or not downgraded for this particular domain  (e.g., inadequate handling of participants who receive a comparator intervention or the duration of follow-up) |
| 79 | Inconsistency rating | 0; 0.5; 1; 2; Not applicable | Did the review authors rate down for inconsistency? |
| 80 | TTE-specific inconsistency | Yes (specify); No; Unclear; Not applicable | Did the review authors consider TTE-specific sources of inconsistency  "Not applicable" = No GRADE rating or not downgraded for this particular domain  (e.g., proportional hazards in some of the included studies and non-proportional in others) |
| 81 | Comments on TTE-specific aspects of the review authors' GRADE rating |  |  |
| 82 | Competing events discussed? | Yes; No; Not applicable | Were competing events mentioned in the discussion? |
| 83 | Competing events discussion |  | Comment on how competing events were discussed |
| 84 | Outcome heterogeneity discussed? | Yes; No; Not applicable | Was heterogeneity between outcome definitions mentioned in the discussion for this outcome? |
| 85 | Outcome heterogeneity discussion |  | Comment on how heterogeneity was discussed |
| 86 | Varying follow-up times discussed? | Yes; No; Not applicable | Were difference in the follow-up times of trials mentioned in the discussion for this outcome? |
| 87 | Follow-up discussion |  | Comment on how varying follow-up among included trials was discussed |
| 88 | Adjusted or unadjusted HRs discussed? | Yes; No; Not applicable | Was the inclusion of adjusted and/or unadjusted hazards ratios in this meta-analysis mentioned in the discussion |
| 89 | Adjusted and unadjusted discussion |  | Comment on how the inclusion of adjusted and/ or unadjusted hazard ratios was discussed |
| 90 | MOD discussed? | Yes; No; Not applicable | Was missing outcome data in the included trials mentioned in the discussion for this outcome? |
| 91 | MOD discussion |  | Comment on how missing outcome data was discussed |
| 92 | Non-administrative censoring discussed? | Yes; No; Not applicable | Was non-administrative censoring (informative censoring) in the included trials mentioned in the discussion for this outcome? |
| 93 | Censoring discussion |  | Comment on whether censoring was discussed, in particular censoring for non-administrative reasons (informative censoring) |
| 94 | Reception of comparator treatments discussed? | Yes; No; Not applicable | Was the reception of comparator treatments in trial participants in the included trials mentioned in the discussion for this outcome? |
| 95 | Comparator treatments discussion |  | Comment on how the reception of comparator treatments was discussed |
| 96 | Other comments |  | Comments on the consideration of time-to-event specific aspects in the review discussion |
| Abbreviations: CI = confidence interval, HR = hazard ratio, ITT, = intention to treat, mITT = modified intention to treat, MOD = missing outcome data, MST = mean survival time, PICO = population-intervention-comparator-outcomes, RoB = risk of bias | | | |

## Appendix A3: Flow-diagram

## Appendix A4: Search results

Between December 2017 and August 2020, we identified 2164 CR (for summary see Flow diagram in A3). After screening titles and abstracts, we assessed the full texts of 74 CR. Fifty finally eligible CR were published from 28/02/2017 to 18/08/2020. For that timeframe, our search strategy identified 2613 records from Core Clinical Journals, of which we selected 401 for full-text screening. Finally, we drew our random sample of 50 nCR from a total sample of 308 eligible reviews.

## Appendix A5: List of included reviews

***Cochrane reviews***

1. Ameratunga M, Pavlakis N, Wheeler H, Grant R, Simes J, Khasraw M. Anti‐angiogenic therapy for high‐grade glioma. Cochrane Database of Systematic Reviews. 2018(11).

2. Arora M, Harvey LA, Glinsky JV, Nier L, Lavrencic L, Kifley A, et al. Electrical stimulation for treating pressure ulcers. Cochrane Database of Systematic Reviews. 2020(1).

3. Bala MM, Celinska‐Lowenhoff M, Szot W, Padjas A, Kaczmarczyk M, Swierz MJ, et al. Antiplatelet and anticoagulant agents for secondary prevention of stroke and other thromboembolic events in people with antiphospholipid syndrome. Cochrane Database of Systematic Reviews. 2017(10).

4. Blank O, von Tresckow B, Monsef I, Specht L, Engert A, Skoetz N. Chemotherapy alone versus chemotherapy plus radiotherapy for adults with early stage Hodgkin lymphoma. Cochrane Database of Systematic Reviews. 2017(4).

5. Bui KT, Willson ML, Goel S, Beith J, Goodwin A. Ovarian suppression for adjuvant treatment of hormone receptor‐positive early breast cancer. Cochrane Database of Systematic Reviews. 2020(3).

6. Bulsara VM, Worthington HV, Glenny AM, Clarkson JE, Conway DI, Macluskey M. Interventions for the treatment of oral and oropharyngeal cancers: surgical treatment. Cochrane Database of Systematic Reviews. 2018(12).

7. Chan DLH, Segelov E, Wong RSH, Smith A, Herbertson RA, Li BT, et al. Epidermal growth factor receptor (EGFR) inhibitors for metastatic colorectal cancer. Cochrane Database of Systematic Reviews. 2017(6).

8. Chin V, Nagrial A, Sjoquist K, O'Connor CA, Chantrill L, Biankin AV, et al. Chemotherapy and radiotherapy for advanced pancreatic cancer. Cochrane Database of Systematic Reviews. 2018(3).

9. Chionh F, Lau D, Yeung Y, Price T, Tebbutt N. Oral versus intravenous fluoropyrimidines for colorectal cancer. Cochrane Database of Systematic Reviews. 2017(7).

10. Claassen YHM, van der Valk MJM, Breugom AJ, Frouws MA, Bastiaannet E, Liefers GJ, et al. Survival differences with immediate versus delayed chemotherapy for asymptomatic incurable metastatic colorectal cancer. Cochrane Database of Systematic Reviews. 2018(11).

11. Coleridge SL, Bryant A, Lyons TJ, Goodall RJ, Kehoe S, Morrison J. Chemotherapy versus surgery for initial treatment in advanced ovarian epithelial cancer. Cochrane Database of Systematic Reviews. 2019(10).

12. Dalal A, Eskin‐Schwartz M, Mimouni D, Ray S, Days W, Hodak E, et al. Interventions for the prevention of recurrent erysipelas and cellulitis. Cochrane Database of Systematic Reviews. 2017(6).

13. Egger SJ, Willson ML, Morgan J, Walker HS, Carrick S, Ghersi D, et al. Platinum‐containing regimens for metastatic breast cancer. Cochrane Database of Systematic Reviews. 2017(6).

14. El Moheb M, Nicolas J, Khamis AM, Iskandarani G, Akl EA, Refaat M. Implantable cardiac defibrillators for people with non‐ischaemic cardiomyopathy. Cochrane Database of Systematic Reviews. 2018(12).

15. Fisher SA, Cutler A, Doree C, Brunskill SJ, Stanworth SJ, Navarrete C, et al. Mesenchymal stromal cells as treatment or prophylaxis for acute or chronic graft‐versus‐host disease in haematopoietic stem cell transplant (HSCT) recipients with a haematological condition. Cochrane Database of Systematic Reviews. 2019(1).

16. Frost JA, Webster KE, Bryant A, Morrison J. Lymphadenectomy for the management of endometrial cancer. Cochrane Database of Systematic Reviews. 2017(10).

17. Galaal K, Donkers H, Bryant A, Lopes AD. Laparoscopy versus laparotomy for the management of early stage endometrial cancer. Cochrane Database of Systematic Reviews. 2018(10).

18. Haun MW, Estel S, Rücker G, Friederich HC, Villalobos M, Thomas M, et al. Early palliative care for adults with advanced cancer. Cochrane Database of Systematic Reviews. 2017(6).

19. Hickey BE, James ML, Daly T, Soh FY, Jeffery M. Hypofractionation for clinically localized prostate cancer. Cochrane Database of Systematic Reviews. 2019(9).

20. Høeg BL, Bidstrup PE, Karlsen RV, Friberg AS, Albieri V, Dalton SO, et al. Follow‐up strategies following completion of primary cancer treatment in adult cancer survivors. Cochrane Database of Systematic Reviews. 2019(11).

21. Hwang EC, Sathianathen NJ, Jung JH, Kim MH, Dahm P, Risk MC. Single‐dose intravesical chemotherapy after nephroureterectomy for upper tract urothelial carcinoma. Cochrane Database of Systematic Reviews. 2019(5).

22. Janmaat VT, Steyerberg EW, van der Gaast A, Mathijssen RHJ, Bruno MJ, Peppelenbosch MP, et al. Palliative chemotherapy and targeted therapies for esophageal and gastroesophageal junction cancer. Cochrane Database of Systematic Reviews. 2017(11).

23. Jeffery M, Hickey BE, Hider PN. Follow‐up strategies for patients treated for non‐metastatic colorectal cancer. Cochrane Database of Systematic Reviews. 2019(9).

24. Jung JH, Risk MC, Goldfarb R, Reddy B, Coles B, Dahm P. Primary cryotherapy for localised or locally advanced prostate cancer. Cochrane Database of Systematic Reviews. 2018(5).

25. Khan L, Soliman H, Sahgal A, Perry J, Xu W, Tsao MN. External beam radiation dose escalation for high grade glioma. Cochrane Database of Systematic Reviews. 2020(5).

26. Kindts I, Laenen A, Depuydt T, Weltens C. Tumour bed boost radiotherapy for women after breast‐conserving surgery. Cochrane Database of Systematic Reviews. 2017(11).

27. Küley‐Bagheri Y, Kreuzer KA, Monsef I, Lübbert M, Skoetz N. Effects of all‐trans retinoic acid (ATRA) in addition to chemotherapy for adults with acute myeloid leukaemia (AML) (non‐acute promyelocytic leukaemia (non‐APL)). Cochrane Database of Systematic Reviews. 2018(8).

28. Kunath F, Jensen K, Pinart M, Kahlmeyer A, Schmidt S, Price CL, et al. Early versus deferred standard androgen suppression therapy for advanced hormone‐sensitive prostate cancer. Cochrane Database of Systematic Reviews. 2019(6).

29. Lee A, Arasaratnam M, Chan DH, Khasraw M, Howell VM, Wheeler H. Anti‐epidermal growth factor receptor therapy for glioblastoma in adults. Cochrane Database of Systematic Reviews. 2020(5).

30. Majumdar A, Roccarina D, Thorburn D, Davidson BR, Tsochatzis E, Gurusamy KS. Management of people with early‐ or very early‐stage hepatocellular carcinoma. Cochrane Database of Systematic Reviews. 2017(3).

31. Morrison J, Thoma C, Goodall RJ, Lyons TJ, Gaitskell K, Wiggans AJ, et al. Epidermal growth factor receptor blockers for the treatment of ovarian cancer. Cochrane Database of Systematic Reviews. 2018(10).

32. Norman G, Christie J, Liu Z, Westby MJ, Jefferies JM, Hudson T, et al. Antiseptics for burns. Cochrane Database of Systematic Reviews. 2017(7).

33. O'Carrigan B, Wong MHF, Willson ML, Stockler MR, Pavlakis N, Goodwin A. Bisphosphonates and other bone agents for breast cancer. Cochrane Database of Systematic Reviews. 2017(10).

34. Pasquali S, Hadjinicolaou AV, Chiarion Sileni V, Rossi CR, Mocellin S. Systemic treatments for metastatic cutaneous melanoma. Cochrane Database of Systematic Reviews. 2018(2).

35. Patil CG, Pricola K, Sarmiento JM, Garg SK, Bryant A, Black KL. Whole brain radiation therapy (WBRT) alone versus WBRT and radiosurgery for the treatment of brain metastases. Cochrane Database of Systematic Reviews. 2017(9).

36. Rai BP, Bondad J, Vasdev N, Adshead J, Lane T, Ahmed K, et al. Robotic versus open radical cystectomy for bladder cancer in adults. Cochrane Database of Systematic Reviews. 2019(4).

37. Rosenberg JE, Jung JH, Edgerton Z, Lee H, Lee S, Bakker CJ, et al. Retzius‐sparing versus standard robotic‐assisted laparoscopic prostatectomy for the treatment of clinically localized prostate cancer. Cochrane Database of Systematic Reviews. 2020(8).

38. Saeaib N, Peeyananjarassri K, Liabsuetrakul T, Buhachat R, Myriokefalitaki E. Hormone replacement therapy after surgery for epithelial ovarian cancer. Cochrane Database of Systematic Reviews. 2020(1).

39. Sathianathen NJ, Philippou YA, Kuntz GM, Konety BR, Gupta S, Lamb AD, et al. Taxane‐based chemohormonal therapy for metastatic hormone‐sensitive prostate cancer. Cochrane Database of Systematic Reviews. 2018(10).

40. Schmidt S, Kunath F, Coles B, Draeger DL, Krabbe LM, Dersch R, et al. Intravesical Bacillus Calmette‐Guérin versus mitomycin C for Ta and T1 bladder cancer. Cochrane Database of Systematic Reviews. 2020(1).

41. Sim EHA, Yang IA, Wood‐Baker R, Bowman RV, Fong KM. Gefitinib for advanced non‐small cell lung cancer. Cochrane Database of Systematic Reviews. 2018(1).

42. Skoetz N, Will A, Monsef I, Brillant C, Engert A, von Tresckow B. Comparison of first‐line chemotherapy including escalated BEACOPP versus chemotherapy including ABVD for people with early unfavourable or advanced stage Hodgkin lymphoma. Cochrane Database of Systematic Reviews. 2017(5).

43. Tosello G, Torloni MR, Mota BS, Neeman T, Riera R. Breast surgery for metastatic breast cancer. Cochrane Database of Systematic Reviews. 2018(3).

44. Tsao MN, Xu W, Wong RKS, Lloyd N, Laperriere N, Sahgal A, et al. Whole brain radiotherapy for the treatment of newly diagnosed multiple brain metastases. Cochrane Database of Systematic Reviews. 2018(1).

45. Vasconcellos VF, Marta GN, da Silva EMK, Gois AFT, de Castria TB, Riera R. Cisplatin versus carboplatin in combination with third‐generation drugs for advanced non‐small cell lung cancer. Cochrane Database of Systematic Reviews. 2020(1).

46. Vellayappan BA, Soon YY, Ku GY, Leong CN, Lu JJ, Tey JCS. Chemoradiotherapy versus chemoradiotherapy plus surgery for esophageal cancer. Cochrane Database of Systematic Reviews. 2017(8).

47. Vernooij RWM, Lancee M, Cleves A, Dahm P, Bangma CH, Aben KKH. Radical prostatectomy versus deferred treatment for localised prostate cancer. Cochrane Database of Systematic Reviews. 2020(6).

48. Vogel N, Schandelmaier S, Zumbrunn T, Ebrahim S, de Boer WEL, Busse JW, et al. Return‐to‐work coordination programmes for improving return to work in workers on sick leave. Cochrane Database of Systematic Reviews. 2017(3).

49. Zaheed M, Wilcken N, Willson ML, O'Connell DL, Goodwin A. Sequencing of anthracyclines and taxanes in neoadjuvant and adjuvant therapy for early breast cancer. Cochrane Database of Systematic Reviews. 2019(2).

50. Zhu J, Li R, Tiselius E, Roudi R, Teghararian O, Suo C, et al. Immunotherapy (excluding checkpoint inhibitors) for stage I to III non‐small cell lung cancer treated with surgery or radiotherapy with curative intent. Cochrane Database of Systematic Reviews. 2017(12).

***Non-Cochrane reviews***

1. Al-Khatib SM, Fonarow GC, Joglar JA, Inoue LYT, Mark DB, Lee KL, et al. Primary Prevention Implantable Cardioverter Defibrillators in Patients With Nonischemic Cardiomyopathy: A Meta-analysis. JAMA Cardiol. 2017;2(6):685-8.

2. Alba AC, Foroutan F, Duero Posada J, Battioni L, Schofield T, Alhussein M, et al. Implantable cardiac defibrillator and mortality in non-ischaemic cardiomyopathy: an updated meta-analysis. Heart. 2018;104(3):230-6.

3. Alnimer Y, Hindi Z, Katato K. The Effect of Perioperative Bevacizumab on Disease-Free and Overall Survival in Locally Advanced HER-2 Negative Breast Cancer: A Meta-Analysis. Breast Cancer. 2018;12:1178223418792250.

4. Arnott C, Li Q, Kang A, Neuen BL, Bompoint S, Lam CSP, et al. Sodium-Glucose Cotransporter 2 Inhibition for the Prevention of Cardiovascular Events in Patients With Type 2 Diabetes Mellitus: A Systematic Review and Meta-Analysis. J Am Heart Assoc. 2020;9(3):e014908.

5. Balitsky AK, Karkar A, McCurdy A, Rochwerg B, Mian HS. Maintenance therapy in transplant ineligible adults with newly-diagnosed multiple myeloma: A systematic review and meta-analysis. Eur J Haematol. 2020;105(5):626-34.

6. Berger MD, Trelle S, Buchi AE, Jegerlehner S, Ionescu C, Lamy de la Chapelle T, et al. Impact on survival through consolidation radiotherapy for diffuse large B-cell lymphoma: a comprehensive meta-analysis. Haematologica. 2020;06:18.

7. Cai W, Yuan Y, Ge W, Fan Y, Liu X, Wu D, et al. EGFR Target Therapy Combined with Gemox for Advanced Biliary Tract Cancers: a Meta-analysis based on RCTs. Journal of Cancer. 2018;9(8):1476-85.

8. Caparica R, Bruzzone M, Hachem GE, Ceppi M, Lambertini M, Glasberg J, et al. Adjuvant chemotherapy in biliary tract cancer patients: A systematic review and meta-analysis of randomized controlled trials. Crit Rev Oncol Hematol. 2020;149:102940.

9. Caparica R, Bruzzone M, Poggio F, Ceppi M, de Azambuja E, Lambertini M. Anthracycline and taxane-based chemotherapy versus docetaxel and cyclophosphamide in the adjuvant treatment of HER2-negative breast cancer patients: a systematic review and meta-analysis of randomized controlled trials. Breast Cancer Res Treat. 2019;174(1):27-37.

10. Changal K, Masroor S, Elzanaty A, Patel M, Mir T, Khan S, et al. Meta-Analysis Comparing Multiple Arterial Grafts Versus Single Arterial Graft for Coronary-Artery Bypass Grafting. Am J Cardiol. 2020;130:46-55.

11. Chen S, Hu B, Li H. A meta-analysis of nivolumab for the treatment of advanced non-small-cell lung cancer. Onco Targets Ther. 2018;11:7691-7.

12. Ciccarese C, Iacovelli R, Bria E, Mosillo C, Bimbatti D, Fantinel E, et al. Second-line therapy for metastatic urothelial carcinoma: Defining the best treatment option among immunotherapy, chemotherapy, and antiangiogenic targeted therapies. A systematic review and meta-analysis. Semin Oncol. 2019;46(1):65-72.

13. Dong YW, Shi YQ, He LW, Cui XY, Su PZ. Effects of metformin on survival outcomes of pancreatic cancer: a meta-analysis. Oncotarget. 2017;8(33):55478-88.

14. Engel S, Awerbuch A, Kwon D, Picado O, Yechieli R, Yakoub D, et al. Optimal radiation dosing in concurrent neoadjuvant chemoradiation for resectable esophageal cancer: a meta-analysis. Journal of Gastrointestinal Oncology. 2020;10(3):391-9.

15. Giacoppo D, Colleran R, Cassese S, Frangieh AH, Wiebe J, Joner M, et al. Percutaneous Coronary Intervention vs Coronary Artery Bypass Grafting in Patients With Left Main Coronary Artery Stenosis: A Systematic Review and Meta-analysis. JAMA Cardiol. 2017;2(10):1079-88.

16. Giugliano D, Maiorino MI, Bellastella G, Longo M, Chiodini P, Esposito K. GLP-1 receptor agonists for prevention of cardiorenal outcomes in type 2 diabetes: An updated meta-analysis including the REWIND and PIONEER 6 trials. Diabetes Obes Metab. 2019;21(11):2576-80.

17. Haller PM, Sulzgruber P, Kaufmann C, Geelhoed B, Tamargo J, Wassmann S, et al. Bleeding and ischaemic outcomes in patients treated with dual or triple antithrombotic therapy: systematic review and meta-analysis. Eur Heart J Cardiovasc Pharmacother. 2019;5(4):226-36.

18. Han D, Wang G, Sun L, Ren X, Shang W, Xu L, et al. Comparison of irinotecan/platinum versus etoposide/platinum chemotherapy for extensive-stage small cell lung cancer: A meta-analysis. Eur J Cancer Care (Engl). 2017;26(6).

19. Han S, Hong Y, Liu T, Wu N, Ye Z. The efficacy and safety of paclitaxel and carboplatin with versus without bevacizumab in patients with non-small-cell lung cancer: a systematic review and meta-analysis. Oncotarget. 2018;9(18):14619-29.

20. Hao C, Tian J, Liu H, Li F, Niu H, Zhu B. Efficacy and safety of anti-PD-1 and anti-PD-1 combined with anti-CTLA-4 immunotherapy to advanced melanoma: A systematic review and meta-analysis of randomized controlled trials. Medicine (Baltimore). 2017;96(26):e7325.

21. Hu H, Zhu Q, Luo XS, Yang XW, Wang HD, Guo CY. Efficacy of PD-1/PD-L1 inhibitors against pretreated advanced cancer: a systematic review and meta-analysis. Oncotarget. 2018;9(14):11846-57.

22. Jang HJ, Kim HS, Kim JH, Lee J. The Effect of Statin Added to Systemic Anticancer Therapy: A Meta-Analysis of Randomized, Controlled Trials. Journal of Clin Med. 2018;7(10):04.

23. Kumar A, Reljic T, Hamadani M, Mohty M, Kharfan-Dabaja MA. Antithymocyte globulin for graft-versus-host disease prophylaxis: an updated systematic review and meta-analysis. Bone Marrow Transplant. 2019;54(7):1094-106.

24. Landre T, Des Guetz G, Chouahnia K, Duchemann B, Assie JB, Chouaid C. First-line angiogenesis inhibitor plus erlotinib versus erlotinib alone for advanced non-small-cell lung cancer harboring an EGFR mutation. J Cancer Res Clin Oncol. 2020;146(12):3333-9.

25. Leal F, Ferreira FP, Sasse AD. FOLFOXIRI Regimen for Metastatic Colorectal Cancer: A Systematic Review and Meta-Analysis. Clin Colorectal Cancer. 2017;16(4):405-9.e2.

26. Liu JW, Chen C, Loh EW, Chu CC, Wang MY, Ouyang HJ, et al. Tyrosine kinase inhibitors for advanced or metastatic thyroid cancer: a meta-analysis of randomized controlled trials. Curr Med Res Opin. 2018;34(5):795-803.

27. Ma H, Wu X, Tao M, Tang N, Li Y, Zhang X, et al. Efficacy and safety of bevacizumab-based maintenance therapy in metastatic colorectal cancer: A meta-analysis. Medicine (Baltimore). 2019;98(50):e18227.

28. Matuschek C, Bolke E, Haussmann J, Mohrmann S, Nestle-Kramling C, Gerber PA, et al. The benefit of adjuvant radiotherapy after breast conserving surgery in older patients with low risk breast cancer- a meta-analysis of randomized trials. Radiat. 2017;12(1):60.

29. Mauri D, Zarkavelis G, Filis P, Tsali L, Zafeiri G, Papadaki A, et al. Postoperative chemotherapy with single-agent fluoropyrimidines after resection of colorectal cancer liver metastases: a meta-analysis of randomised trials. ESMO open. 2018;3(4):e000343.

30. Miyashita M, Hattori M, Takano T, Toyama T, Iwata H. Risks and benefits of bevacizumab combined with chemotherapy for advanced or metastatic breast cancer: a meta-analysis of randomized controlled trials. Breast Cancer. 2020;27(3):347-54.

31. Montagnani F, Di Leonardo G, Pino M, Perboni S, Ribecco A, Fioretto L. Protracted Inhibition of Vascular Endothelial Growth Factor Signaling Improves Survival in Metastatic Colorectal Cancer: A Systematic Review. Journal of Translational Internal Medicine. 2017;5(1):18-26.

32. Natori A, Ethier JL, Amir E, Cescon DW. Capecitabine in early breast cancer: A meta-analysis of randomised controlled trials. Eur J Cancer. 2017;77:40-7.

33. Ottaiano A, Capozzi M, De Divitiis C, De Stefano A, Botti G, Avallone A, et al. Gemcitabine mono-therapy versus gemcitabine plus targeted therapy in advanced pancreatic cancer: a meta-analysis of randomized phase III trials. Acta Oncol. 2017;56(3):377-83.

34. Palmerini T, Serruys P, Kappetein AP, Genereux P, Riva DD, Reggiani LB, et al. Clinical outcomes with percutaneous coronary revascularization vs coronary artery bypass grafting surgery in patients with unprotected left main coronary artery disease: A meta-analysis of 6 randomized trials and 4,686 patients. Am Heart J. 2017;190:54-63.

35. Poggio F, Ceppi M, Lambertini M, Bruzzi P, Ugolini D, Bighin C, et al. Concurrent versus sequential adjuvant chemo-endocrine therapy in hormone-receptor positive early stage breast cancer patients: a systematic review and meta-analysis. Breast. 2017;33:104-8.

36. Pula A, Stawiski K, Braun M, Iskierka-Jazdzewska E, Robak T. Efficacy and safety of B-cell receptor signaling pathway inhibitors in relapsed/refractory chronic lymphocytic leukemia: a systematic review and meta-analysis of randomized clinical trials. Leuk Lymphoma. 2018;59(5):1084-94.

37. Ramos-Esquivel A, van der Laat A, Rojas-Vigott R, Juarez M, Corrales-Rodriguez L. Anti-PD-1/anti-PD-L1 immunotherapy versus docetaxel for previously treated advanced non-small cell lung cancer: a systematic review and meta-analysis of randomised clinical trials. ESMO open. 2017;2(3):e000236.

38. Raphael J, Chan K, Karim S, Kerbel R, Lam H, Santos KD, et al. Antiangiogenic Therapy in Advanced Non-small-cell Lung Cancer: A Meta-analysis of Phase III Randomized Trials. Clin Lung Cancer. 2017;18(4):345-53.e5.

39. Reljic T, Kumar A, Klocksieben FA, Djulbegovic B. Treatment targeted at underlying disease versus palliative care in terminally ill patients: a systematic review. BMJ Open. 2017;7(1):e014661.

40. Roviello G, Corona SP, D'Angelo A, Rosellini P, Nobili S, Mini E. Immune Checkpoint Inhibitors in Pre-Treated Gastric Cancer Patients: Results from a Literature-Based Meta-Analysis. Int J Molec Sc. 2020;21(2):10.

41. Schmitt AM, Herbrand AK, Fox CP, Bakunina K, Bromberg JEC, Cwynarski K, et al. Rituximab in primary central nervous system lymphoma-A systematic review and meta-analysis. Hematol Oncol. 2019;16:16.

42. Shui L, Wu YS, Lin H, Shui P, Sun Q, Chen X. Triplet Chemotherapy (FOLFOXIRI) Plus Bevacizumab Versus Doublet Chemotherapy (FOLFOX/FOLFIRI) Plus Bevacizumab in Conversion Therapy for Metastatic Colorectal Cancer: a Meta-Analysis. Cell Physiol Biochem. 2018;48(5):1870-81.

43. Stavrakis S, Asad Z, Reynolds D. Implantable Cardioverter Defibrillators for Primary Prevention of Mortality in Patients With Nonischemic Cardiomyopathy: A Meta-Analysis of Randomized Controlled Trials. J Cardiovasc Electrophysiol. 2017;28(6):659-65.

44. Tang X, He J, Li B, Zheng Y, Li K, Zou S, et al. Efficacy and Safety of Gefitinib in Patients with Advanced Head and Neck Squamous Cell Carcinoma: A Meta-Analysis of Randomized Controlled Trials. J Oncol. 2019;2019:6273438.

45. Tringali A, Hassan C, Rota M, Rossi M, Mutignani M, Aabakken L. Covered vs. uncovered self-expandable metal stents for malignant distal biliary strictures: a systematic review and meta-analysis. Endoscopy. 2018;50(6):631-41.

46. Vidal L, Gurion R, Shargian L, Dreyling M, Gafter-Gvili A. Bendamustine for patients with indolent B cell lymphoproliferative malignancies including chronic lymphocytic leukaemia - an updated meta-analysis. Br J Haematol. 2019;186(2):234-42.

47. Wang J, Xu B, Wang W, Zhai X, Chen X. Efficacy and safety of fulvestrant in postmenopausal patients with hormone receptor-positive advanced breast cancer: a systematic literature review and meta-analysis. Breast Cancer Res Treat. 2018;171(3):535-44.

48. Wang X, Bao Z, Zhang X, Li F, Lai T, Cao C, et al. Effectiveness and safety of PD-1/PD-L1 inhibitors in the treatment of solid tumors: a systematic review and meta-analysis. Oncotarget. 2017;8(35):59901-14.

49. Xu L, Yan N, Li Z, Luo L, Wu X, Liu Q, et al. A comparison of fulvestrant plus a targeted agent with fulvestrant alone in hormone receptor-positive advanced breast cancer that progressed on prior endocrine therapy: a meta-analysis. Onco Targets Ther. 2018;11:8389-98.

50. Zhong S, Qie S, Yang L, Yan Q, Ge L, Wang Z. S-1 monotherapy versus S-1 combination therapy in gemcitabine-refractory advanced pancreatic cancer: A meta-analysis (PRISMA) of randomized control trials. Medicine (Baltimore). 2017;96(30):e7611.

Appendix-Table A6: Extended characteristics of reviews

| **Category** | **Item** | **Review** | | | **Review time-to-event outcomes** | | |
| --- | --- | --- | --- | --- | --- | --- | --- |
|  |  | **Overall  (N = 100)** | **Cochrane  (n = 50)** | **Non-Cochrane  (n = 50)** | **Overall (N = 217)** | **Cochrane  (n = 93)** | **Non-Cochrane  (n = 124)** |
| **Publication** | | | | | | | |
| *Publication year* | 2017 | 36% (36) | 36% (18) | 36% (18) |  |  |  |
|  | 2018 | 28% (28) | 28% (14) | 28% (14) |  |  |  |
|  | 2019 | 18% (18) | 18% (9) | 18% (9) |  |  |  |
|  | 2020 | 18% (18) | 18% (9) | 18% (9) |  |  |  |
| *Journal Impact Factor* | Median (IQR) |  | 11.87 | 4.41 (3.33 - 6.18) |  |  |  |
|  | Mean (Range) |  | 11.87 | 6.37 (1.817 - 35.86) |  |  |  |
| *Review update* | Yes | 27% (27) | 50% (25) | 4% (2) |  |  |  |
| *Multiple review comparisons* | Yes | 28% (28) | 44% (22) | 12% (6) |  |  |  |
| **Population** | | | | | | | |
| *Medical field* | Neoplasms | 82% (82) | 86% (43) | 78% (39) |  |  |  |
|  | Diseases of the circulatory system | 11% (11) | 4% (2) | 18% (9) |  |  |  |
|  | Diseases of the skin and subcutaneous tissue | 3% (3) | 6% (3) | 0% (0) |  |  |  |
|  | Diseases of blood, -forming organs and immune mechanism | 2% (2) | 2% (1) | 2% (1) |  |  |  |
|  | Other | 2% (2) | 2% (1) | 2% (1) |  |  |  |
| *Medical condition* | Breast cancer | 13% (13) | 10% (5) | 16% (8) |  |  |  |
|  | Colorectal cancer | 9% (9) | 8% (4) | 10% (5) |  |  |  |
|  | Non-small cell lung cancer | 8% (8) | 6% (3) | 10% (5) |  |  |  |
|  | Prostate cancer | 6% (6) | 12% (6) | 0% (0) |  |  |  |
|  | Non-ischemic cardiomyopathy | 4% (4) | 2% (1) | 6% (3) |  |  |  |
|  | Other | 3% (3) | 60% (30) | 58% (29) |  |  |  |
| *Clinical stage* | Advanced/ Second or third line | 36% (36) | 30% (15) | 42% (21) |  |  |  |
|  | Early/ First line | 30% (30) | 38% (19) | 22% (11) |  |  |  |
|  | No restriction | 12% (12) | 16% (8) | 8% (4) |  |  |  |
|  | Not reported | 13% (13) | 10% (5) | 16% (8) |  |  |  |
|  | Not applicable | 9% (9) | 6% (3) | 12% (6) |  |  |  |
| *Age group* | Adults | 96% (96) | 98% (49) | 94% (47) |  |  |  |
|  | Both | 1% (1) | 2% (1) | 0% (0) |  |  |  |
|  | Not reported | 3% (3) | 0% (0) | 6% (3) |  |  |  |
| **Interventions** | | | | | | | |
| *Experimental intervention* | Biologics/ drug | 58% (58) | 40% (20) | 76% (38) |  |  |  |
|  | Surgical procedure | 10% (10) | 14% (7) | 6% (3) |  |  |  |
|  | Radiation | 5% (5) | 10% (5) | 0% (0) |  |  |  |
|  | Biologics/ drug, Surgical procedure | 4% (4) | 6% (3) | 2% (1) |  |  |  |
|  | Medical devices | 4% (4) | 0% (0) | 8% (4) |  |  |  |
|  | Other | 3% (3) | 30% (15) | 8% (4) |  |  |  |
| *Comparator intervention* | Biologics/ drugs | 42% (42) | 28% (14) | 56% (28) |  |  |  |
|  | Surgical procedure | 9% (9) | 8% (4) | 10% (5) |  |  |  |
|  | Best supportive/ Optimal medical care | 7% (7) | 8% (4) | 6% (3) |  |  |  |
|  | Observation | 5% (5) | 2% (1) | 8% (4) |  |  |  |
|  | Placebo, No treatment | 5% (5) | 10% (5) | 0% (0) |  |  |  |
|  | Radiation | 5% (5) | 10% (5) | 0% (0) |  |  |  |
|  | Other | 28% (28) | 36% (18) | 20% (10) |  |  |  |
| *Comparisons* | Biologics/ drugs | 37% (37) | 24% (12) | 50% (25) |  |  |  |
|  | Surgical procedures | 7% (7) | 8% (4) | 6% (3) |  |  |  |
|  | Biologics/ drug vs. Best supportive/ Optimal medical care | 4% (4) | 4% (2) | 4% (2) |  |  |  |
|  | Biologics/ drug vs. Observation | 4% (4) | 0% (0) | 8% (4) |  |  |  |
|  | Biologics/ drug vs. Biologics/ Drugs, Placebo | 3% (3) | 2% (1) | 4% (2) |  |  |  |
|  | Biologics/ drug vs. Placebo | 3% (3) | 0% (0) | 6% (3) |  |  |  |
|  | Biologics/ drug vs. Placebo, No treatment | 3% (3) | 6% (3) | 0% (0) |  |  |  |
|  | Radiation | 3% (3) | 6% (3) | 0% (0) |  |  |  |
|  | Biologics/ drug (schedule alteration) | 2% (2) | 2% (1) | 2% (1) |  |  |  |
|  | Follow-up strategies | 2% (2) | 4% (2) | 0% (0) |  |  |  |
|  | Other | 32% (32) | 44% (22) | 20% (10) |  |  |  |
| *Comparator treatment considered?* | Yes | 2% (2) | 2% (1) | 2% (1) |  |  |  |
| **Outcomes - Planned** | | | | | | | |
| *Planned outcome number* | Median (IQR) | 5 (4 - 8) | 7 (5 - 8) | 4 (3 - 5) |  |  |  |
|  | Mean (Range) | 5.79 (1 - 15) | 6.82 (3 - 12) | 4.67 (1 - 15) |  |  |  |
| *Planned TTE outcome number* | Median | 2 (2 - 2) | 2 (2 - 3) | 2 (2 - 2) |  |  |  |
|  | Mean | 2.39 (1 - 12) | 2.17 (1 - 4) | 2.62 (1 - 12) |  |  |  |
| *Planned TTE outcomes* | Overall survival, ACM or death from any cause | 89% (89) | 88% (44) | 90% (45) |  |  |  |
|  | Progression-free survival | 44% (44) | 36% (18) | 52% (26) |  |  |  |
|  | Disease-free survival | 13% (13) | 16% (8) | 10% (5) |  |  |  |
|  | Myocardial infarction | 5% (5) | 0% (0) | 10% (5) |  |  |  |
|  | Stroke | 5% (5) | 0% (0) | 10% (5) |  |  |  |
|  | Cardiovascular mortality | 4% (4) | 2% (1) | 6% (3) |  |  |  |
|  | Time to death from prostate cancer | 4% (4) | 8% (4) | 0% (0) |  |  |  |
|  | Cardiac death | 4% (4) | 2% (1) | 6% (3) |  |  |  |
|  | Other | 3% (3) | 62% (31) | 58% (29) |  |  |  |
|  | Unclear | 2% (2) | 2% (1) | 2% (1) |  |  |  |
|  | Not reported | 1% (1) | 0% (0) | 2% (1) |  |  |  |
|  | Not applicable | 2% (2) | 2% (1) | 2% (1) |  |  |  |
| *Number of outcomes analyzed* | Median | 5 (3 - 6) | 5 (4 - 6.75) | 4 (2.25 - 5) |  |  |  |
|  | Mean | 4.83 (1 - 12) | 5.34 (1 - 12) | 4.32 (1 - 12) |  |  |  |
| *Number of TTE outcomes analyzed* | Median | 2 (1 - 2) | 2 (1 - 2) | 2 (2 - 2) |  |  |  |
|  | Mean | 2.23 (1 - 12) | 1.92 (1 - 4) | 2.54 (1 - 12) |  |  |  |
| *TTE outcomes in methods different from analyzed* | Yes | 11% (11) | 20% (10) | 2% (1) |  |  |  |
| *Reasons for difference in mentioned and analyzed TTE outcomes* | Outcome not assessed in trial(s) | 7% (7) | 14% (7) | 0% (0) |  |  |  |
|  | Time-to-event data not available in trial(s) | 2% (2) | 2% (1) | 0% (0) |  |  |  |
|  | Not pooled due to clinical heterogeneity | 1% (1) | 2% (1) | 0% (0) |  |  |  |
|  | Not reported | 1% (1) | 0% (0) | 2% (1) |  |  |  |
|  | Not applicable | 89% (89) | 80% (40) | 98% (49) |  |  |  |
| *TTE outcomes analyzed* | Overall survival, ACM or death from any cause | 89% (89) | 84% (42) | 94% (47) | 41% (89) | 45% (42) | 38% (47) |
|  | Progression-free survival | 39% (39) | 26% (13) | 52% (26) | 18% (39) | 14% (13) | 21% (26) |
|  | Disease-free survival | 10% (10) | 10% (5) | 10% (5) | 5% (10) | 5% (5) | 4% (5) |
|  | Myocardial infarction | 6% (6) | 0% (0) | 12% (6) | 3% (6) | 0% (0) | 5% (6) |
|  | Stroke | 5% (5) | 0% (0) | 10% (5) | 2% (5) | 0% (0) | 4% (5) |
|  | Cardiac death | 4% (4) | 2% (1) | 6% (3) | 2% (4) | 1% (1) | 2% (3) |
|  | Cardiovascular mortality | 3% (3) | 2% (1) | 4% (2) | 1% (3) | 1% (1) | 2% (2) |
|  | Major adverse cardiac events (MACE) | 3% (3) | 0% (0) | 6% (3) | 1% (3) | 0% (0) | 2% (3) |
|  | Other | 3% (3) | 62% (31) | 54% (27) | 1% (3) | 33% (31) | 22% (27) |
| **Sample size** | | | | | | | |
| *Number of included studies in reviews and meta-analyses* | Median | 5 (4 - 8) | 5 (3 - 10) | 6 (4 - 8) | 4 (3 - 7) | 4 (2 - 6) | 5 (4 - 7) |
|  | Mean | 6.69 (2 - 24) | 7.12 (2 - 24) | 6.26 (2 - 19) | 5.25 (2 - 19) | 5.05 (2 - 19) | 5.40 (2 - 19) |
| *Total population in review or meta-analysis* | Median | 1722 (978 - 4390) | 1415 (572 - 4022) | 1866 (1395 - 4526) | 811 (308 - 2876) | 711 (177 - 2327) | 1042 (698 - 3173) |
|  | Mean | 3877 (170 - 56004) | 2795 (170 - 13216) | 4911 (343 - 56004) | 5745 (181 - 38723) | 2656 (181 - 13949) | 8985 (482 - 38723) |
|  | Not reported | 12% (12) | 14% (7) | 10% (5) | 23% (49) | 8% (7) | 34% (42) |
| *Experimental population in review or meta-analysis* | Median | 765 (373 - 1539) | 223 (159 - 641) | 937 (645 - 1804) | 791 (290 - 2197) | 715 (181 - 1772) | 981 (645 - 3076) |
|  | Mean | 1235 (90 - 5039) | 402 (90 - 951) | 1529 (238 - 5039) | 1840 (81 - 12373) | 1722.64 (81 - 12373) | 1963 (238 - 5039) |
|  | Not reported | 77% (77) | 88% (44) | 66% (33) | 62% (135) | 55% (51) | 68% (84) |
| *Control population in review or meta-analysis* | Median | 765 (373 - 1539) | 223 (159 - 641) | 937 (645 - 1804) | 2025 (879 - 4822) | 1407 (446 - 3657) | 4394 (1580 - 9302) |
|  | Mean | 1128 (80 - 4278) | 432 (80 - 1145) | 1374 (244 - 4278) | 1575 (80 - 6403) | 1377 (80 - 6403) | 1783 (244 - 4278) |
|  | Not reported | 77% (77) | 88% (44) | 66% (33) | 62% (135) | 55% (51) | 68% (84) |
| Abbreviations: ACM = all-cause mortality, TTE = time to event | | | | | | | |

Appendix-Table A7: Extended characteristics of included time-to-event review outcomes

| **Category** | **Item** |  | **Review** |  | **Review time-to-event outcomes** | | | | | |
| --- | --- | --- | --- | --- | --- | --- | --- | --- | --- | --- |
|  |  |  |  |  |  | **Overall** |  | **All-cause mortality/ Overall survival (n = 89)** | **Combined, including all-cause mortality (n = 63)** | **Not including all-cause mortality (n = 65)** |
|  |  | **Overall  (N = 100)** | **Cochrane  (n = 50)** | **Non-Cochrane  (n = 50)** | **Overall (N = 217)** | **Cochrane  (n = 93)** | **Non-Cochrane  (n = 124)** |  |  |  |
| *TTE outcomes as primary outcomes* | Yes | 69% (69) | 92% (46) | 46% (23) | 39% (85) | 62% (58) | 22% (27) | 55% (49) | 29% (18) | 28% (18) |
|  | No | 3% (3) | 6% (3) | 0% (0) | 33% (72) | 37% (34) | 31% (38) | 16% (14) | 41% (26) | 49% (32) |
|  | Not applicable | 28% (28) | 2% (1) | 54% (27) | 28% (60) | 1% (1) | 48% (59) | 29% (26) | 30% (19) | 23% (15) |
| *TTE outcomes that were primary outcomes* | Overall survival, ACM or death from any cause | 71% (49) | 76% (35) | 61% (14) | 58% (49) | 60% (35) | 52% (14) | 100% (49) | 0% (0) | 0% (0) |
|  | Progression-free survival | 20% (14) | 13% (6) | 35% (8) | 16% (14) | 10% (6) | 30% (8) | 0% (0) | 71% (12) | 11% (2) |
|  | Disease-free survival | 6% (4) | 7% (3) | 4% (1) | 5% (4) | 5% (3) | 4% (1) | 0% (0) | 24% (4) | 0% (0) |
|  | Time to complete healing | 3% (2) | 4% (2) | 0% (0) | 2% (2) | 3% (2) | 0% (0) | 0% (0) | 0% (0) | 11% (2) |
|  | All-cause death, myocardial infarction or stroke | 1% (1) | 0% (0) | 4% (1) | 1% (1) | 0% (0) | 4% (1) | 0% (0) | 6% (1) | 0% (0) |
|  | Any thromboembolic event | 1% (1) | 2% (1) | 0% (0) | 1% (1) | 2% (1) | 0% (0) | 0% (0) | 0% (0) | 5% (1) |
|  | Cardiac death | 1% (1) | 2% (1) | 0% (0) | 1% (1) | 2% (1) | 0% (0) | 0% (0) | 6% (1) | 0% (0) |
|  | Cardiovascular mortality | 1% (1) | 2% (1) | 0% (0) | 1% (1) | 2% (1) | 0% (0) | 0% (0) | 0% (0) | 5% (1) |
|  | Other | 1% (1) | 20% (9) | 13% (3) | 1% (1) | 16% (9) | 11% (3) | 0% (0) | 0% (0) | 5% (1) |
| *Reviews providing definitions of TTE outcomes* | For all outcomes | 48% (48) | 74% (37) | 22% (11) |  |  |  |  |  |  |
|  | For ≥1 outcome | 7% (7) | 6% (3) | 8% (4) |  |  |  |  |  |  |
|  | For no outcome | 45% (45) | 20% (10) | 70% (35) |  |  |  |  |  |  |
| *TTE outcome definition per outcome* | Yes |  |  |  | 48% (104) | 83% (77) | 22% (27) | 51% (45) | 56% (35) | 37% (24) |
|  | No |  |  |  | 52% (113) | 17% (16) | 78% (97) | 49% (44) | 44% (28) | 63% (41) |
| *Composite TTE outcomes* | Yes | 39% (39) | 42% (21) | 36% (18) | 22% (47) | 24% (22) | 20% (25) | 0% (0) | 56% (35) | 18% (12) |
|  | No | 97% (97) | 98% (49) | 96% (48) | 65% (140) | 69% (64) | 61% (76) | 100% (89) | 0% (0) | 78% (51) |
|  | Unclear/ Not reported | 29% (29) | 12% (6) | 46% (23) | 14% (30) | 8% (7) | 19% (23) | 0% (0) | 44% (28) | 3% (2) |
| *Composite events described* | Yes | 87% (34) | 90% (19) | 83% (15) | 87% (41) | 91% (20) | 84% (21) | 0% (NA) | 91% (32) | 75% (9) |
| *All-cause mortality part of outcome definition* | Yes | 90% (90) | 86% (43) | 94% (47) | 57% (124) | 65% (60) | 52% (64) | 100% (89) | 56% (35) | 0% (0) |
|  | No | 29% (29) | 40% (20) | 18% (9) | 29% (62) | 28% (26) | 29% (36) | 0% (0) | 0% (0) | 95% (62) |
|  | Unclear | 31% (31) | 14% (7) | 48% (24) | 14% (31) | 8% (7) | 19% (24) | 0% (0) | 44% (28) | 5% (3) |
| *Death as competing event possible* | Yes | 29% (29) | 40% (20) | 18% (9) | 29% (64) | 28% (26) | 31% (38) | 1% (1) | 2% (1) | 98% (64) |
|  | No | 90% (90) | 86% (43) | 94% (47) | 56% (121) | 62% (58) | 51% (63) | 99% (88) | 52% (33) | 186% (121) |
|  | Unclear | 31% (31) | 16% (8) | 46% (23) | 15% (32) | 10% (9) | 19% (23) | 0% (0) | 46% (29) | 49% (32) |
| *Reviews reporting outcomes as events of absence of events* | Absence of event only | 61% (61) | 48% (24) | 74% (37) |  |  |  |  |  |  |
|  | Event only | 24% (24) | 26% (13) | 22% (11) |  |  |  |  |  |  |
|  | Both (with reasoning) | 5% (5) | 10% (5) | 0% (0) |  |  |  |  |  |  |
|  | Mixed | 2% (2) | 4% (2) | 0% (0) |  |  |  |  |  |  |
|  | At least one unclear (without reasoning) | 8% (8) | 12% (6) | 4% (2) |  |  |  |  |  |  |
| *Outcome reporting as events or absence of event* | Absence of event |  |  |  | 54% (118) | 53% (49) | 56% (69) | 71% (63) | 81% (51) | 6% (4) |
|  | Event |  |  |  | 37% (81) | 30% (28) | 43% (53) | 19% (17) | 13% (8) | 86% (56) |
|  | Both (with reasoning) |  |  |  | 5% (10) | 11% (10) | 0% (0) | 7% (6) | 2% (1) | 5% (3) |
|  | Unclear (both without reasoning) |  |  |  | 4% (8) | 6% (6) | 2% (2) | 3% (3) | 5% (3) | 3% (2) |
| *Reviews including follow-up start in outcome definitions* | Randomization | 32% (32) | 48% (24) | 16% (8) |  |  |  |  |  |  |
|  | Allocated treatment | 2% (2) | 2% (1) | 2% (1) |  |  |  |  |  |  |
|  | Enrollment | 2% (2) | 2% (1) | 0% (0) |  |  |  |  |  |  |
|  | Mixed for different outcomes | 2% (2) | 2% (1) | 0% (0) |  |  |  |  |  |  |
|  | At least one not applicable (e.g., start not reported) | 62% (62) | 46% (23) | 82% (41) |  |  |  |  |  |  |
| *Follow-up start included in outcome definition* | Randomization |  |  |  | 32% (70) | 57% (53) | 14% (17) | 37% (33) | 33% (21) | 25% (16) |
|  | Enrollment |  |  |  | 3% (6) | 6% (6) | 0% (0) | 4% (4) | 3% (2) | 0% (0) |
|  | Allocated treatment |  |  |  | 2% (4) | 2% (2) | 2% (2) | 1% (1) | 2% (1) | 3% (2) |
|  | Multiple time points (e.g., "enrollment or treatment") |  |  |  | 1% (2) | 2% (2) | 0% (0) | 1% (1) | 2% (1) | 0% (0) |
|  | Not applicable (e.g., start of follow-up not reported) |  |  |  | 62% (135) | 32% (30) | 85% (105) | 56% (50) | 60% (38) | 72% (47) |
| Abbreviations: ACM = all-cause mortality, TTE = time to event | | | | | | | | | | |

Appendix-Table A8: Analysis principles and adjustment status of trial analyses included in time-to-event meta-analyses of included reviews

| **Category** | **Item** | **Review** | | | **Review time-to-event outcomes** | | |
| --- | --- | --- | --- | --- | --- | --- | --- |
|  |  | **Overall  (N = 100)** | **Cochrane  (n = 50)** | **Non-Cochrane  (n = 50)** | **Overall (N = 217)** | **Cochrane  (n = 93)** | **Non-Cochrane  (n = 124)** |
| *Types of analyses eligible in reviews* | ITT | 42% (42) | 72% (36) | 12% (6) |  |  |  |
|  | Not reported | 58% (58) | 28% (14) | 88% (44) |  |  |  |
| *Types of analyses eligible for outcome analyses* | ITT | 1% (1) | 0% () | 2% (1) | 1% (2) | 0% (0) | 2% (2) |
|  | Not reported | 99% (99) | 100% (50) | 98% (49) | 99% (215) | 100% (93) | 98% (122) |
| *Types of analyses included in reviews* | ITT | 21% (21) | 24% (12) | 18% (9) |  |  |  |
|  | Included trial(s) did not report type of analysis | 6% (6) | 10% (5) | 2% (1) |  |  |  |
|  | mITT | 4% (4) | 8% (4) | 0% (0) |  |  |  |
|  | PP | 2% (2) | 4% (2) | 0% (0) |  |  |  |
|  | Other | 2% (2) | 4% (2) | 0% (0) |  |  |  |
|  | Not reported for all trials | 16% (16) | 28% (14) | 4% (2) |  |  |  |
|  | Not reported for any trial | 63% (63) | 48% (24) | 78% (39) |  |  |  |
| *Types of analyses included in outcome analyses* | ITT | 2% (2) | 2% (1) | 2% (1) | 2% (5) | 2% (2) | 2% (3) |
|  | Not reported for all trials | 1% (1) | 2% (1) | 0% (0) | 0% (1) | 1% (1) | 0% (0) |
|  | Not reported | 97% (97) | 96% (48) | 98% (49) | 97% (211) | 97% (90) | 98% (121) |
| *Unadjusted/ adjusted HRs eligible in reviews* | Both | 4% (4) | 8% (4) | 0% (0) |  |  |  |
|  | Hierarchical (adjusted before unadjusted) | 4% (4) | 6% (3) | 2% (1) |  |  |  |
|  | Unadjusted only | 2% (2) | 2% (1) | 2% (1) |  |  |  |
|  | Adjusted only | 1% (1) | 2% (1) | 0% (0) |  |  |  |
|  | Hierarchical (unadjusted before adjusted) | 1% (1) | 2% (1) | 0% (0) |  |  |  |
|  | Unclear | 6% (6) | 10% (5) | 2% (1) |  |  |  |
|  | Not reported | 82% (82) | 90% (45) | 94% (47) |  |  |  |
| *Dealing with unadjusted/ adjusted HRs* | Included in interpretation of heterogeneity | 2% (2) | 4% (2) | 0% () |  |  |  |
|  | Combined in meta-analysis | 1% (1) | 0% () | 2% (1) |  |  |  |
|  | Unclear | 7% (7) | 12% (6) | 2% (1) |  |  |  |
|  | Not reported | 8% (8) | 14% (7) | 2% (1) |  |  |  |
|  | Not applicable (unadjusted/ adjusted not mentioned) | 82% (82) | 70% (35) | 94% (47) |  |  |  |
| *Stratified HRs eligible in reviews* | Yes | 1% (1) | 2% (1) | 0% (0) |  |  |  |
|  | Unclear | 1% (1) | 2% (1) | 0% (0) |  |  |  |
|  | No | 98% (98) | 96% (48) | 100% (50) |  |  |  |
| *Unadjusted/ adjusted HRs eligible in outcome analyses* | Unadjusted only | 2% (2) | 4% (2) | 0% (0) | 1% (2) | 2% (2) | 0% (0) |
|  | Adjusted only | 1% (1) | 2% (1) | 0% (0) | 0% (1) | 1% (1) | 0% (0) |
|  | Not reported | 97% (97) | 94% (47) | 100% (50) | 99% (214) | 97% (90) | 100% (124) |
| *Dealing with unadjusted/ adjusted HRs* | Only unadjusted/adjusted HRs included in analysis | 2% (2) | 4% (2) | 0% (0) | 1% (2) | 2% (2) | 0% (0) |
|  | Unadjusted HRs recalculated | 1% (1) | 2% (1) | 0% (0) | 0% (1) | 1% (1) | 0% (0) |
|  | Not applicable | 97% (97) | 94% (47) | 100% (50) | 99% (214) | 97% (90) | 100% (124) |
| *Unadjusted/ adjusted HRs discussed in reviews* | In discussions | 2% (2) | 2% (1) | 2% (1) |  |  |  |
|  | In results | 1% (1) | 2% (1) | 0% (0) |  |  |  |
|  | Not applicable | 1% (1) | 0% (0) | 2% (1) |  |  |  |
|  | Not reported | 96% (96) | 96% (48) | 96% (48) |  |  |  |
| *Unadjusted/ adjusted in discussion for individual outcome* | No | 100% (100) | 100% (50) | 100% (50) | 100% (217) | 100% (93) | 100% (124) |
| Abbreviations: HR = hazard ratio, ITT = intention to treat, mITT = modified intention to treat | | | | | | | |

Appendix-Table A9: Extended information on time-to-event specific methods and time-to-event data acquirement in included reviews

| **Category** | **Item** |  | **Review** |  | **Review time-to-event outcomes** | | | | | |
| --- | --- | --- | --- | --- | --- | --- | --- | --- | --- | --- |
|  |  |  |  |  |  | **Overall** |  | **All-cause mortality/ Overall survival (n = 89)** | **Combined, including all-cause mortality (n = 63)** | **Not including all-cause mortality (n = 65)** |
|  |  | **Overall  (N = 100)** | **Cochrane  (n = 50)** | **Non-Cochrane  (n = 50)** | **Overall (N = 217)** | **Cochrane  (n = 93)** | **Non-Cochrane  (n = 124)** |  |  |  |
| *HR type eligible in reviews* | HR/ log(HR) not further specified | 91% (91) | 94% (47) | 88% (44) |  |  |  |  |  |  |
|  | Cox model HR/ log(HR) | 2% (2) | 4% (2) | 0% (0) |  |  |  |  |  |  |
|  | Cox model HR, log-rank test and KM-Curve | 1% (1) | 2% (1) | 0% (0) |  |  |  |  |  |  |
|  | Not reported | 6% (6) | 0% (0) | 12% (6) |  |  |  |  |  |  |
| *HR types eligible per outcome* | HR/ log(HR) from Cox model | 1% (1) | 2% (1) | 0% (0) | 0% (1) | 1% (1) | 0% (0) | 1% (1) | 0% (0) | 0% (0) |
|  | HR/ log(HR) from median survival times and CI | 1% (1) | 2% (1) | 0% (0) | 0% (1) | 1% (1) | 0% (0) | 1% (1) | 0% (0) | 0% (0) |
|  | Unclear | 1% (1) | 2% (1) | 0% (0) | 1% (3) | 3% (3) | 0% (0) | 1% (1) | 0% (0) | 3% (2) |
|  | Not reported | 97% (97) | 94% (47) | 100% (50) | 98% (212) | 95% (88) | 100% (124) | 97% (86) | 100% (63) | 97% (63) |
| *Methods to obtain TTE data per review* | HR and confidence intervals | 64% (64) | 66% (33) | 62% (31) |  |  |  |  |  |  |
|  | Specified set of methods (e.g., Tierney 2008) | 46% (46) | 76% (38) | 16% (8) |  |  |  |  |  |  |
|  | log(HR) and standard error | 16% (16) | 26% (13) | 6% (3) |  |  |  |  |  |  |
|  | Survival curves | 13% (13) | 14% (7) | 12% (6) |  |  |  |  |  |  |
|  | HR with other information (e.g., events) | 10% (10) | 16% (8) | 4% (2) |  |  |  |  |  |  |
|  | P-value with other information (e.g., events) | 8% (8) | 8% (4) | 8% (4) |  |  |  |  |  |  |
|  | IPD (recalculated or from publication) | 4% (4) | 4% (2) | 4% (2) |  |  |  |  |  |  |
|  | Median survival times | 4% (4) | 8% (4) | 0% (0) |  |  |  |  |  |  |
|  | Other (Formular by Parmar, not TTE specific) | 1% (1) | 0% (0) | 2% (1) |  |  |  |  |  |  |
|  | Time point specific survival times | 1% (1) | 2% (1) | 0% (0) |  |  |  |  |  |  |
|  | Risk ratio | 1% (1) | 0% (0) | 2% (1) |  |  |  |  |  |  |
|  | Unclear | 6% (6) | 8% (4) | 4% (2) |  |  |  |  |  |  |
|  | Not reported | 16% (16) | 0% (0) | 32% (16) |  |  |  |  |  |  |
| *Recalculation of TTE data reported for an outcome* | Yes | 18% (18) | 34% (17) | 2% (1) |  |  |  |  |  |  |
| *Methods to obtain TTE data for an outcome* | HR and confidence intervals | 9% (9) | 18% (9) | 0% (0) | 7% (16) | 17% (16) | 0% (0) | 9% (8) | 5% (3) | 8% (5) |
|  | P-value with other information (e.g., events) | 4% (4) | 8% (4) | 0% (0) | 5% (10) | 11% (10) | 0% (0) | 4% (4) | 5% (3) | 5% (3) |
|  | Survival curves | 7% (7) | 12% (6) | 2% (1) | 5% (10) | 10% (9) | 1% (1) | 6% (5) | 2% (1) | 6% (4) |
|  | HR with other information (e.g., events) | 1% (1) | 2% (1) | 0% (0) | 1% (3) | 3% (3) | 0% (0) | 1% (1) | 2% (1) | 2% (1) |
|  | RevMan calculator | 1% (1) | 2% (1) | 0% (0) | 1% (3) | 3% (3) | 0% (0) | 1% (1) | 2% (1) | 2% (1) |
|  | Time point specific survival times | 1% (1) | 2% (1) | 0% (0) | 1% (2) | 2% (2) | 0% (0) | 1% (1) | 0% (0) | 2% (1) |
|  | Trial exclusion due to inability to recalculate data | 1% (1) | 2% (1) | 0% (0) | 0% (1) | 1% (1) | 0% (0) | 0% (0) | 2% (1) | 0% (0) |
|  | IPD (recalculated or from publication) | 1% (1) | 2% (1) | 0% (0) | 0% (1) | 1% (1) | 0% (0) | 1% (1) | 0% (0) | 0% (0) |
|  | Median survival times | 1% (1) | 2% (1) | 0% (0) | 0% (1) | 1% (1) | 0% (0) | 1% (1) | 0% (0) | 0% (0) |
|  | Unclear | 5% (5) | 10% (5) | 0% (0) | 3% (6) | 6% (6) | 0% (0) | 4% (4) | 0% (0) | 3% (2) |
| Abbreviations: CI = confidence intervals, HR = hazard ratio; KM = Kaplan-Meier, TTE = time-to-event | | | | | | | | | | |

Appendix-Table A10: Handling of specific trial characteristics with relevance for time-to-event outcomes in the included reviews

| **Category** | **Item** | **Review** | | | **Review time-to-event outcomes** | | |
| --- | --- | --- | --- | --- | --- | --- | --- |
|  |  | **Overall  (N = 100)** | **Cochrane  (n = 50)** | **Non-Cochrane  (n = 50)** | **Overall (N = 217)** | **Cochrane  (n = 93)** | **Non-Cochrane  (n = 124)** |
| **Heterogeneous outcome definitions of included trials** | | | | | | | |
| *Reviews mentioning heterogeneous TTE outcome definitions* | In discussion | 3% (3) | 0% (0) | 6% (3) |  |  |  |
|  | In results | 3% (3) | 6% (3) | 0% (0) |  |  |  |
|  | In results and discussion | 1% (1) | 2% (1) | 0% (0) |  |  |  |
|  | Not reported | 93% (93) | 92% (46) | 94% (47) |  |  |  |
| *Heterogeneous outcome definitions discussed* | Yes | 4% (4) | 4% (2) | 4% (2) | 2% (4) | 2% (2) | 2% (2) |
| **Follow-up** | | | | | | | |
| *Reviews reporting a planned follow-up duration* | Minimum duration of follow-up required | 5% (5) | 6% (3) | 4% (2) |  |  |  |
|  | Longest follow-up | 4% (4) | 4% (2) | 4% (2) |  |  |  |
|  | Maximum duration of follow-up specified | 2% (2) | 4% (2) | 0% (0) |  |  |  |
|  | Time-specific (12 months, 2 year, 10 year, ...) | 1% (1) | 2% (1) | 0% (0) |  |  |  |
|  | Not reported | 88% (88) | 84% (42) | 92% (46) |  |  |  |
| *Follow-up time specification for TTE outcomes* | Longest follow-up | 8% (8) | 8% (4) | 8% (4) | 13% (29) | 9% (8) | 17% (21) |
|  | Minimum duration of follow-up required | 2% (2) | 4% (2) | 0% (0) | 3% (6) | 6% (6) | 0% (0) |
|  | Maximum duration of follow-up specified | 3% (3) | 4% (2) | 2% (1) | 1% (3) | 2% (2) | 1% (1) |
|  | "Time-specific (12 months, 2 year, 10 year, ...)" | 2% (2) | 4% (2) | 0% (0) | 1% (2) | 2% (2) | 0% (0) |
|  | Not reported | 85% (85) | 80% (40) | 90% (45) | 82% (177) | 81% (75) | 82% (102) |
| **Analyses - Varying follow-up between included trials** | | | | | | | |
| *Dealing with varying follow-up in reviews* | Sensitivity analyses (e.g., shorter/longer follow-up) | 8% (8) | 14% (7) | 2% (1) |  |  |  |
|  | Included in interpretation of heterogeneity | 2% (2) | 4% (2) | 0% (0) |  |  |  |
|  | Included in meta-regression | 2% (2) | 0% (0) | 4% (2) |  |  |  |
|  | Excluded studies with divergent follow-up time | 1% (1) | 0% (0) | 2% (1) |  |  |  |
|  | Follow-up time for comparisons pre-determined | 1% (1) | 2% (1) | 0% (0) |  |  |  |
|  | Mentioned as RoB criterion in methods | 1% (1) | 2% (1) | 0% (0) |  |  |  |
|  | Pre-defined timing as inclusion criterion | 1% (1) | 0% (0) | 2% (1) |  |  |  |
|  | Unclear | 1% (1) | 0% (0) | 2% (1) |  |  |  |
|  | Not reported | 83% (83) | 78% (39) | 88% (44) |  |  |  |
| *Dealing with varying follow-up for individual outcomes* | Included in meta-regression | 2% (2) | 2% (1) | 2% (1) | 1% (2) | 1% (1) | 1% (1) |
|  | Results reported for multiple time-points | 1% (1) | 2% (1) | 0% (0) | 0% (1) | 1% (1) | 0% (0) |
|  | Not reported per outcome | 97% (97) | 96% (48) | 98% (49) | 99% (214) | 98% (91) | 99% (123) |
| *Varying follow-up discussed* | In discussions | 10% (10) | 10% (5) | 10% (5) |  |  |  |
|  | In results | 7% (7) | 10% (5) | 4% (2) |  |  |  |
|  | In results and discussion | 5% (5) | 8% (4) | 2% (1) |  |  |  |
|  | Not reported | 77% (77) | 70% (35) | 84% (42) |  |  |  |
|  | Not applicable | 1% (1) | 2% (1) | 0% (0) |  |  |  |
| *Varying follow-up in discussion for individual outcomes* | Yes | 5% (5) | 4% (2) | 6% (3) | 3% (7) | 4% (4) | 2% (3) |
|  | No | 94% (94) | 94% (47) | 94% (47) | 96% (209) | 95% (88) | 98% (121) |
|  | Not applicable | 1% (1) | 2% (1) | 0% (0) | 0% (1) | 1% (1) | 0% (0) |
| **Analyses - Missing outcome data** | | | | | | | |
| *Dealing with missing outcome data in reviews* | Mentioned as RoB criterion in methods | 71% (71) | 94% (47) | 48% (24) |  |  |  |
|  | Contact with authors | 8% (8) | 14% (7) | 2% (1) |  |  |  |
|  | Sensitivity analyses (according to rate of MOD) | 7% (7) | 14% (7) | 0% (0) |  |  |  |
|  | Single imputation | 4% (4) | 8% (4) | 0% (0) |  |  |  |
|  | Not reported | 26% (26) | 0% (0) | 52% (26) |  |  |  |
| *Dealing with missing data in individual outcomes* | Single imputation | 1% (1) | 2% (1) | 0% (0) | 0% (1) | 1% (1) | 0% (0) |
|  | Not reported per outcome | 99% (99) | 98% (49) | 100% (50) | 100% (216) | 99% (92) | 100% (124) |
| *Missing outcome data discussed* | In results | 59% (59) | 78% (39) | 40% (20) |  |  |  |
|  | In results and discussion | 6% (6) | 12% (6) | 0% (0) |  |  |  |
|  | Not reported | 35% (35) | 10% (5) | 60% (30) |  |  |  |
| *Missing outcome data included in discussion for individual outcomes* | Yes | 2% (2) | 4% (2) | 0% (0) | 1% (2) | 2% (2) | 0% (0) |
|  | No | 98% (98) | 96% (48) | 100% (50) | 99% (215) | 98% (91) | 100% (124) |
| **Analyses - Informative censoring** | | | | | | | |
| *Dealing with informative censoring in reviews* | Mentioned as RoB criterion in methods | 3% (3) | 6% (3) | 0% (0) |  |  |  |
|  | Not reported | 97% (97) | 94% (47) | 100% (50) |  |  |  |
| *Dealing with informative censoring in individual outcomes* | Not reported per outcome | 100% (100) | 100% (50) | 100% (50) | 0% (217) | 100% (93) | 100% (124) |
| *Informative censoring discussed* | In discussion | 1% (1) | 2% (1) | 0% (0) |  |  |  |
|  | In results | 1% (1) | 2% (1) | 0% (0) |  |  |  |
|  | Not reported | 98% (98) | 96% (48) | 100% (50) |  |  |  |
| *Informative censoring in discussion for individual outcomes* | No | 100% (100) | 100% (50) | 100% (50) | 0% (217) | 100% (93) | 100% (124) |
| **Analyses - Competing events** | | | | | | | |
| *Dealing with (*deaths as) *competing event in reviews* | Mentioned as RoB criterion in methods | 1% (1) | 0% (0) | 2% (1) |  |  |  |
|  | Not reported | 59% (59) | 58% (29) | 60% (30) |  |  |  |
|  | Not applicable | 40% (40) | 42% (21) | 38% (19) |  |  |  |
| *Dealing with (deaths as) competing events in individual outcomes* | Not reported per outcome | 60% (60) | 58% (29) | 62% (31) | 48% (105) | 46% (43) | 50% (62) |
|  | Not applicable | 40% (40) | 42% (21) | 38% (19) | 52% (112) | 54% (50) | 50% (62) |
| *(Deaths as) Competing events discussed* | In results and in discussion | 1% (1) | 0% () | 2% (1) |  |  |  |
|  | Not reported | 59% (59) | 58% (29) | 60% (30) |  |  |  |
|  | Not applicable | 40% (40) | 42% (21) | 38% (19) |  |  |  |
| *(Deaths as) Competing events included in discussion for individual outcomes* | Yes | 1% (1) | 2% (1) | 0% (0) | 0% (1) | 1% (1) | 0% (0) |
|  | No | 59% (59) | 56% (28) | 62% (31) | 48% (104) | 45% (42) | 50% (62) |
|  | Not applicable | 40% (40) | 42% (21) | 38% (19) | 52% (112) | 54% (50) | 50% (62) |
| **Analyses - Treatment switching** | | | | | | | |
| *Dealing with treatment switching in reviews* | Mentioned as RoB criterion in methods | 4% (4) | 4% (2) | 4% (2) |  |  |  |
|  | Complies with review PICO | 2% (2) | 4% (2) | 0% (0) |  |  |  |
|  | Presence reported for each trial | 2% (2) | 4% (2) | 0% (0) |  |  |  |
|  | Sensitivity analysis (e.g., according to rate) | 2% (2) | 2% (1) | 2% (1) |  |  |  |
|  | Sensitivity analysis (e.g., as treated trial data) | 1% (1) | 0% (0) | 2% (1) |  |  |  |
|  | Not reported | 91% (91) | 88% (44) | 94% (47) |  |  |  |
| *Dealing with treatment switching in individual outcomes* | Not reported per outcome | 100% (100) | 100% (50) | 100% (50) | 0% (217) | 100% (93) | 100% (124) |
| *Treatment switching discussed* | In discussions | 5% (5) | 6% (3) | 4% (2) |  |  |  |
|  | In results | 5% (5) | 8% (4) | 2% (1) |  |  |  |
|  | In results and discussion | 2% (2) | 2% (1) | 2% (1) |  |  |  |
|  | Not reported | 88% (88) | 84% (42) | 92% (46) |  |  |  |
| *Treatment switching included in discussion for individual outcomes* | Yes | 3% (3) | 2% (1) | 4% (2) | 1% (3) | 1% (1) | 2% (2) |
|  | No | 97% (97) | 98% (49) | 96% (48) | 99% (214) | 99% (92) | 98% (122) |
| **Analyses - Proportional hazards** | | | | | | | |
| *Proportional hazards assessed in reviews* | Not reported | 100% (100) | 100% (50) | 100% (50) |  |  |  |
| *Proportional hazards assessed in individual outcomes* | Not reported per outcome | 100% (100) | 100% (50) | 100% (50) | 0% (217) | 100% (93) | 100% (124) |
| *Dealing with (non-)proportional hazards* | Not applicable | 100% (100) | 100% (50) | 100% (50) |  |  |  |
| *Test for proportionality for individual outcomes* | Not applicable | 100% (100) | 100% (50) | 100% (50) | 0% (217) | 100% (93) | 100% (124) |
| *Non-proportionality of hazards indicated* | Not applicable | 100% (100) | 100% (50) | 100% (50) | 0% (217) | 100% (93) | 100% (124) |
| *Dealing with (non-) proportional hazards* | Not applicable | 100% (100) | 100% (50) | 100% (50) | 0% (217) | 100% (93) | 100% (124) |
| **Risk of Bias** | | | | | | | |
| *Risk of bias tools specified* | Risk of Bias 1 (study level) | 55% (55) | 58% (29) | 52% (26) |  |  |  |
|  | Risk of Bias 1 (outcome level) | 23% (23) | 42% (21) | 4% (2) |  |  |  |
|  | Other (e.g., CONSORT, MERGE) | 6% (6) | 0% (0) | 12% (6) |  |  |  |
|  | Jadad scale | 4% (4) | 0% (0) | 8% (4) |  |  |  |
|  | Risk of Bias 2.0 | 3% (3) | 0% (0) | 6% (3) |  |  |  |
|  | No assessment | 9% (9) | 0% (0) | 18% (9) |  |  |  |
| *TTE specific risk of bias criteria used* | Yes (e.g., "risk of bias related to censoring") | 4% (4) | 8% (4) | 0% (0) |  |  |  |
|  | No | 9% (9) | 92% (46) | 82% (41) |  |  |  |
|  | Not applicable | 87% (87) | 0% (0) | 18% (9) |  |  |  |
| Abbreviations: TTE = time to event, RoB = risk of bias | | | | | | | |

Appendix-Table A11: Absolute effects presented in reviews for time-to-event meta-analyses

| **Category** | **Item** | **Review** | | | **Review time-to-event outcomes** | | |
| --- | --- | --- | --- | --- | --- | --- | --- |
|  |  | **Overall  (N = 100)** | **Cochrane  (n = 50)** | **Non-Cochrane  (n = 50)** | **Overall (N = 217)** | **Cochrane  (n = 93)** | **Non-Cochrane  (n = 124)** |
| *Absolute effects reported* | Yes | 44% (44) | 80% (40) | 8% (4) | 40% (86) | 80% (74) | 10% (12) |
|  | No | 50% (50) | 10% (5) | 90% (45) | 57% (123) | 13% (12) | 90% (111) |
|  | Explicitly not calculated (e.g., " because TTE outcome") | 6% (6) | 10% (5) | 2% (1) | 4% (8) | 8% (7) | 1% (1) |
| *Type of absolute effects* | Natural frequencies | 25% (25) | 46% (23) | 4% (2) | 22% (48) | 42% (39) | 7% (9) |
|  | Risk difference | 13% (13) | 24% (12) | 2% (1) | 13% (28) | 29% (27) | 1% (1) |
|  | Median survival or difference in median survival | 4% (4) | 8% (4) | 0% (0) | 3% (7) | 8% (7) | 0% (0) |
|  | Natural frequencies and risk difference | 2% (2) | 2% (1) | 2% (1) | 1% (3) | 1% (1) | 2% (2) |
|  | Not applicable | 56% (56) | 20% (10) | 92% (46) | 60% (131) | 20% (19) | 90% (112) |
| *Baseline risk applicable for events or absence of events* | Event | 26% (26) | 48% (24) | 4% (2) | 24% (51) | 52% (48) | 2% (3) |
|  | Absence of event | 6% (6) | 12% (6) | 0% (0) | 6% (12) | 13% (12) | 0% (0) |
|  | Unclear | 8% (8) | 14% (7) | 2% (1) | 4% (9) | 9% (8) | 1% (1) |
|  | Not applicable | 60% (60) | 26% (13) | 94% (47) | 67% (145) | 27% (25) | 97% (120) |
| *Description of outcome adapted to direction of baseline risk* | No (no changes) | 32% (32) | 60% (30) | 4% (2) | 26% (57) | 59% (55) | 2% (2) |
|  | Yes (description changed with reasoning) | 8% (8) | 14% (7) | 0% (0) | 6% (12) | 13% (12) | 0% (0) |
|  | Yes (description changed without reasoning) | 2% (2) | 2% (1) | 2% (1) | 2% (4) | 2% (2) | 2% (2) |
|  | Unclear | 1% (1) | 2% (1) | 0% (0) | 1% (3) | 3% (3) | 0% (0) |
|  | Not applicable | 57% (57) | 22% (11) | 94% (47) | 65% (141) | 23% (21) | 97% (120) |
| *Absolute effects correctly calculated* | Yes | 22% (22) | 40% (20) | 4% (2) | 19% (41) | 41% (38) | 2% (3) |
|  | Correct calculation but wrong labeling | 5% (5) | 10% (5) | 0% (0) | 5% (11) | 12% (11) | 0% (0) |
|  | No | 6% (6) | 12% (6) | 0% (0) | 6% (12) | 13% (12) | 0% (0) |
|  | Unclear (e.g., applicability of baseline risk or HR unclear) | 7% (7) | 12% (6) | 2% (1) | 4% (8) | 8% (7) | 1% (1) |
|  | Not applicable | 60% (60) | 26% (13) | 94% (47) | 67% (145) | 27% (25) | 97% (120) |
| Abbreviations: TTE = time to event | | | | | | | |
